# Illness perceptions and explanatory models of viral hepatitis B & C among immigrants and refugees: a narrative systematic review

**DOI:** 10.1186/s12889-015-1476-0

**Published:** 2015-02-15

**Authors:** John A Owiti, Trisha Greenhalgh, Lorna Sweeney, Graham R Foster, Kamaldeep S Bhui

**Affiliations:** Wolfson Institute of Preventive Medicine, Barts and The London School of Medicine and Dentistry, Charterhouse Square, Queen Mary University of London, Centre for Psychiatry, EC1M 6BQ London, UK; Blizard Institute, Barts and The London School of Medicine and Dentistry, Queen Mary University of London, Centre for Primary Care and Public Health, Yvonne Carter Building, 58 Turner Street, E1 2AB London, UK; Blizard Institute, Barts and The London School of Medicine and Dentistry, Queen Mary University of London, The Liver Unit, Centre for Digestive Diseases, 4 Newark Street, E1 2AT London, UK

**Keywords:** Hepatitis B, Hepatitis C, Local knowledge, Perceptions, Attitudes, Migrants, Immigrants

## Abstract

**Background:**

Hepatitis B and C (HBV, HCV) infections are associated with high morbidity and mortality. Many countries with traditionally low prevalence (such as UK) are now planning interventions (screening, vaccination, and treatment) of high-risk immigrants from countries with high prevalence. This review aimed to synthesise the evidence on immigrants’ knowledge of HBV and HCV that might influence the uptake of clinical interventions. The review was also used to inform the design and successful delivery of a randomised controlled trial of targeted screening and treatment.

**Methods:**

Five databases (PubMed, CINHAL, SOCIOFILE, PsycINFO & Web of Science) were systematically searched, supplemented by reference tracking, searches of selected journals, and of relevant websites. We aimed to identify qualitative and quantitative studies that investigated knowledge of HBV and HCV among immigrants from high endemic areas to low endemic areas. Evidence, extracted according to a conceptual framework of Kleinman’s explanatory model, was subjected to narrative synthesis. We adapted the PEN-3 model to categorise and analyse themes, and recommend strategies for interventions to influence help-seeking behaviour.

**Results:**

We identified 51 publications including quantitative (n = 39), qualitative (n = 11), and mixed methods (n = 1) designs. Most of the quantitative studies included small samples and had heterogeneous methods and outcomes. The studies mainly concentrated on hepatitis B and ethnic groups of South East Asian immigrants residing in USA, Canada, and Australia. Many immigrants lacked adequate knowledge of aetiology, symptoms, transmission risk factors, prevention strategies, and treatment, of hepatitis HBV and HCV. Ethnicity, gender, better education, higher income, and English proficiency influenced variations in levels and forms of knowledge.

**Conclusion:**

Immigrants are vulnerable to HBV and HCV, and risk life-threatening complications from these infections because of poor knowledge and help-seeking behaviour. Primary studies in this area are extremely diverse and of variable quality precluding meta-analysis. Further research is needed outside North America and Australia.

**Electronic supplementary material:**

The online version of this article (doi:10.1186/s12889-015-1476-0) contains supplementary material, which is available to authorized users.

## Background

Hepatitis B (HBV) and C (HCV) virus infections are major threats to global public health currently associated with increasing chronic morbidity and mortality rates worldwide [1-3]. Chronic HBV and HCV infections are responsible for nearly 57% of liver cirrhosis cases (HBV 30%, HCV 27%) and 78% of hepatocellular carcinomas (HBV 53%, HCV 25%), the third commonest cause of cancer deaths worldwide [4,5].

HBV is highly endemic in developing regions such as South East Asia, China, Africa, the Middle East and the Amazon Basin, where at least 8% of the population are HBV chronic carriers [3] (Table 1). HBV is moderately endemic in parts of Eastern and Southern Europe, the Middle East, central Asia, South Asia, and parts of South America [3]. The endemicity of HBV is low in most developed areas, such as North America, Northern and Western Europe and Australia [3].

**Table 1:** **Regional prevalence of viral hepatitis B and C**

**Prevalence of chronic HBV across the world**		**Prevalence of chronic HCV across the world**	
**Prevalence**	**Areas of the world**	**Prevalence**	**Areas of the world**
High prevalence >8%	All of Africa; South East Asia (China, Korea, Indonesia & the Philippines); The Middle East, except Israel; South and Western Pacific Islands; The interior Amazon basin; and certain parts of the Caribbean (Haiti and Dominican Republic)	High prevalence >10%	Egypt
Intermediate prevalence 2% - 7%	South Central and Southwest ;Israel; Japan; Eastern and Southern Europe; Russia; Most areas surrounding the Amazon basin; Honduras; and Guatemala	Intermediate prevalence >2%	Countries in Latin America, Eastern Europe, and many countries in Africa, the Middle East, and South Asia
Low prevalence <2%	Northern and Western Europe; North America; Australia; New Zealand ; Mexico; Southern South America	Low prevalence <2%	Northern and Western Europe; North America Australia; New Zealand

Adapted from: *Centers for Disease Control and Prevention, 2014* [6].

In developing countries, HBV is often transmitted vertically from infected mothers to their offspring, horizontally in early childhood through exposure to infected children, donor blood transmission, unsafe therapeutic injection practices (and other healthcare-related procedures), or through sex [7-9]. The main routes for transmission of HCV are not clear but unsafe therapeutic injection and medical practices and donor blood are mentioned [9,10]. The emergence of injecting drug users in settings where the prevalence of HCV is high (Africa, the Middle East and South East Asia) presents an additional threat [9].

The unprecedented rate of migration from high to low prevalence countries may explain the increasing incidence of chronic HBV and HCV infections and mortality rates from hepatocellular carcinoma in North America and Western European countries [11-16]. The proportion of all immigrants chronically infected with HBV range from 3.7% to 9.7% in the different migrant-receiving countries [17]. Compared with indigenous populations, higher rates of chronic viral hepatitis infections, mortality, and morbidity from hepatocellular carcinoma have been found among ethnic immigrants in the Netherlands [18] UK [10,19,20], Australia [21], USA [22], Canada [14], and France [23].

In the UK, over 95% of new chronic HBV infections occur in immigrant populations [24]. Higher rates of chronic HBV infections were found in immigrant women attending antenatal screening in England [25,26]. In another study of 15 specialist hepatic medicine centres in the UK, 81% of the 1,147 patients with chronic HBV registered [27]. The UK’s Chief Medical Officer’s annual report [28] highlighted undiagnosed viral hepatitis infection, predominantly in immigrants, as one of the three major causes of liver disease, contributing to the dramatic rise in liver cancer deaths in people under the age of 65 years.

However, immigrants and refugees from intermediate and high prevalence countries are not routinely screened for HBV and HCV infections, nor is hepatitis B vaccination routinely given in most migrant-receiving countries [29]. Recent guidelines from the United States, Canada, the European Union, and Australia identify immigrants originating from areas of intermediate and high endemicity as an at risk group for HBV who should be screened routinely and vaccinated for HBV [24,29-33]. The World Health Organisation’s ‘call to action’ demands an immediate increase in prevention, screening, and treatment of HBV and HCV infections [34]. In contrast, there is little public or policy awareness of the health implications of chronic HBV and HCV infections across the European Union [35] and immigrants are neither routinely screened nor vaccinated. In the UK, though a national screening programme for HBV and HCV does not exist [36], even though the National Institute of Health and Clinical Excellence has issued guidance for systematic screening in first and second-generation immigrants from known countries of high prevalence [24].

In response to this, a cross-sectional cluster randomised controlled clinical trial of targeted screening and treatment through primary care (GP practices) in England (London, Bradford, and Oxford) called the *HepFree Trial* (UKCRN ID No. 14034) is currently examining the effectiveness and cost-effectiveness among high-risk immigrants.

This narrative systematic review aimed to synthesise evidence on knowledge (illness perceptions or explanatory models) of HBV and HCV infections among first and second-generation migrants from high or intermediate prevalence countries to traditionally low prevalence countries.

## Methods

### Inclusion criteria

Studies were included if they:

(i) used qualitative, quantitative or mixed methods to examine knowledge of, and beliefs about, HBV and/or HCV infections;
(ii) were carried out among first (foreign-born) and second generation (descendants of at least one foreign born) immigrant groups from countries of high prevalence (Sub-Saharan Africa, North Africa, Middle East, Asia, Latin America) or intermediate (Southern and Eastern Europe) prevalence of HBV and/or HCV infections who migrated to traditionally low prevalence countries (North America, Western Europe, Australia, and New Zealand);
(iii) were published in English in peer reviewed journals;
(iv) examined the participants’ experiences with HBV and/or HCV infections and/or with screening, vaccination and treatment; and
(v) were studies of mixed ‘low’ , ‘intermediate’ and ‘high’ risk populations, and it was possible to attribute the findings to specific immigrant population of interest as defined above.

Studies that focussed on injecting drug users were only included if the participants were first- and second-generation migrants and refugees, and if they assessed knowledge and attitudes of participants. Intervention studies on HBV and/or HCV infections and associated liver cancer, were included if they assessed people’s knowledge, attitudes, or behaviour both before and after the intervention.

### Exclusion criteria

Studies were excluded if they focussed on:

(i) the general (non-immigrant) population groups;
(ii) groups at low risk of HBV and HCV infections; and
(iii) other populations at high risk such as injecting drug users, prisoners, and men who have sex with men.

### Search and selection

#### Search strategy

We developed an inclusive search strategy incorporating qualitative and quantitative studies. Seven electronic databases (PubMed, CINHAL, SOCIOFILE, PsycINFO, Web of Science databases (Science Citation Index Expanded (1970-present) Social Sciences Citation Index (1970-present), Arts & Humanities Citation Index (1975-present), Conference Proceedings Citation Index- Science (1990-present), Conference Proceedings Citation Index- Social Science & Humanities (1990-present)), were independently searched by two people in January 2013, and updated in February 2014. The initial search used the terms: ‘belief’ OR ‘perception’ OR ‘culture’ OR ‘Knowledge’ OR ‘practices’ OR ‘narrative’ OR ‘explanatory model’ OR ‘awareness’ OR ‘views’ OR ‘reasons’ OR ‘perspective*’ OR ‘attitude*’ OR ‘motiv*’ AND ‘viral hepatitis’ OR ‘hepatitis B’ OR ‘hepatitis C’ OR ‘HBV’ OR ‘HCV’ OR ‘blood borne virus’ OR ‘liver infection’ OR ‘liver disease’ OR ‘hepatocellular carcinoma’ OR’ liver cancer’ OR ‘jaundice’. The search was then limited by restricting it to those references containing ‘refugee* OR ‘marginal group’ OR ‘minorit*’ OR ‘disadvantaged group’ OR ‘asylum seeker’ OR ‘migrant’ OR ‘immigrant*’.

We also searched web-based sources (e.g. Google scholar, NHS Evidence collection on ethnicity and health, Allied and Complementary Medicine Database (AMED), Health Management Information Consortium (HMIC) and Project MUSE). The following journals were hand searched: Journal of Immigrant and Minority Health, Journal of Community Health, Australian Health Review, Gastroenterology Nursing, International Journal of Drug Policy, Journal of Health Care for the Poor and the Underserved, and Journal of Viral Hepatitis. All sources were subjected to forward and backward citation tracking. Review articles were searched through the JBI register and Cochrane Database for Systematic Reviews.

#### Selection

**Figure 1:**
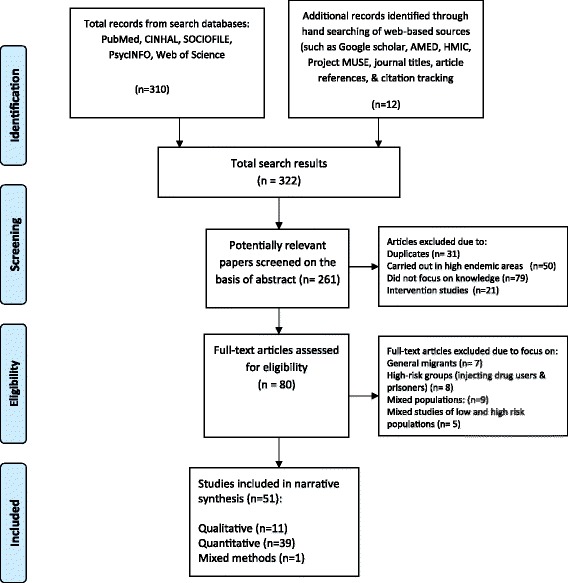
**PRISMA Flowchart of search and studies selection.**

All hits were initially considered for relevance on the basis of the title and abstract. Irrelevant studies were systematically excluded (based on exclusion and inclusion criteria) as detailed in the PRISMA search strategy flow chart (Figure 1). Full text articles were obtained for all those that seemed to meet inclusion criteria, and where it was not possible to make a confident judgement on the basis of the title and abstract. Full articles were further assessed for eligibility following the inclusions and exclusion criteria.

### Data analysis

#### Appraisal of the studies

We used Mixed Methods Appraisal Tool (MMAT) - Version 2011, a scoring system for appraising the methodological quality of qualitative, quantitative, and mixed methods studies [37,38]. Two people (JAO and KSB) appraised the articles independently and compared the outcomes. Any disagreement was resolved through discussion and consensus. We did not exclude any study published in a peer-reviewed journal on the basis of appraisal as some studies that were classed as low methodological quality (for example on the basis of small sample size) contributed to the overall findings and to the overall aim of identifying varied forms of knowledge. We did, however, use consensus when deciding whether a finding reported in a study was valid. Heterogeneity of study design, populations and instruments precluded formal meta-analysis of quantitative data.

#### Data extraction

Two people independently extracted data from the articles. Study details including location, aims, demographic data, study design, sample, and data on knowledge of HBV and HCV infections were tabulated. The latter findings were extracted, by interrogating each study using the conceptual framework of explanatory model [39] as presented by the theoretical framework used.

#### Theoretical framework

We used Kleinman’s explanatory model as our theoretical framework [39]. The explanatory model of an illness or infection offers conceptual and culturally-specific frameworks for understanding how different cultural and social contexts affect the ways that people understand, and negotiate their experiences with illness or infections. The following are the main elements of an explanatory model of illness: aetiology, symptoms, pathophysiology, history and severity (course), and treatment [39,40]. We modified the explanatory model framework to accommodate forms of knowledge applicable to communicable infections such as transmission factors and preventive methods. We were also interested in people’s experiences with these infections, including screening, vaccination, and treatment. We extracted data on the knowledge of HBC and HCV infections and organised them around the following themes: concepts (identity) of HBV and HCV; signs and symptoms; causes; transmission; prevention; consequences; and treatment.

#### Synthesis

We synthesised outcomes from relevant articles using the process and techniques of narrative synthesis approach to develop a robust understanding of knowledge of HBV and HCV infections. Narrative synthesis is useful in synthesising heterogeneous evidence of different types (qualitative and quantitative) [41,42]. Data were initially extracted and grouped into tables according to methods, theoretical frameworks, and focus of studies. All data (from quantitative and qualitative sources) were integrated for complementarity [43,44] and to improve on depth of understanding of the level, and type, of knowledge of HBV and HCV infections. In fact, a mixed-methods systematic narrative review has parallels to the process of triangulation or cross-validation in data synthesis [45]. Survey studies provided quantified levels of knowledge of HBV and HCV in scores (as a % of the correct answer), and these have been pooled together (with low scores indicating a knowledge deficit), but could not be statistically combined. All data were interrogated, interpreted, and organised into a priori thematic categories or elements of the explanatory model, as outlined in the theoretical framework.

We have also adapted the PEN-3 model to further categorise and analyse themes, and to demonstrate specific strategies and approaches for specific areas of interventions to influence health-seeking behaviour. PEN-3 model was originally developed to situate culture at the centre of health-seeking behaviour in health promotion and disease prevention [46-48], and emphasises the meeting and working with beliefs of participants rather than only aiming to change them.

## Results

Two hundred and sixty one studies were screened as potentially relevant out of which 80 full text articles were fully assessed for eligibility according to inclusion and exclusion criteria (Figure 1). We identified 51 relevant peer-reviewed journal articles, published in English [49-99] (Additional file 1: Table S1). In our appraisal of methodological quality, most surveys (n = 20) and qualitative studies (n = 9) had 2 and 3 star ratings (out of a maximum of 4 stars) respectively (Table 2). Salient issues that affected the quality of the studies included inadequate reporting of research methods, sampling methods, unclear research context, lack of reporting of response rate, and low response rates (<60%). Only 19 survey studies reported response rates of more than 60%.

**Table 2:** **Quality ratings for studies**

	1 star (25%)*	**2 stars (50%)**	**3 stars (75%)**	**4 stars (100%)**
Survey studies (n=)	2	20	14	2
Qualitative studies (n=)		2	9	
Mixed methods (n=)		1		

*Key - scores: 1 star (25%) one criterion met; 2 stars (50%) 2 criteria met; 3 stars (75%) 3 criteria met; and 4 stars (100%) met all 4 criteria.

Most studies (64%) had been conducted in the USA (Table 3). Most were cross-sectional surveys that focussed on HBV infection (84%) (Table 4), and drew their samples from the general immigrant populations in the community (n = 37, 72.5%) (Table 5). One online survey included some open-ended questions [53]. One mixed methods study had community-based participants, and identified those with chronic HBV infection for in-depth interviews [69]. Three studies were carried out with participants with chronic HBV infection, and one was conducted among injecting drug users with HCV infection.

**Table 3:** **Location of studies by country**

**Countries**	**Number of studies**	**Sources**
USA	32	[48-52,54,57-59,66,67,69,71-75,77-88,95,96,98]
Australia	8	[53,60,61,64,65,76,93,94]
Canada	5	[56,63,70,89,97]
Netherlands	3	[90-92]
Canada and USA	1	[55]
Mexico and USA	1	[62]

**Table 4:** **Methods used in studies**

**Methods of studies**	**Focus**	**Number of studies**	**Sources**
Cross-sectional surveys	HBV	34	[48,51,55,56,58,59,63,66,68-75,78-89,92,93,95,97,98]
	HCV	2	[61,65]
	HBV & HCV	3	[53,62,76]
	HBV & Liver cancer	2	[57,96]
Qualitative studies	HBV	5	[50,54,67,90,94]
	HCV	1	[60]
	HBV and HCV	1	[64]
	HBV & liver cancer	2	[49,77]
Mixed methods	HBV	1	[52]

**Table 5:** **Participants in, and focus of, studies**

**Type of participants**	**Number of studies**	**Focus**	**Source**
General populations	46	HBV	[48-51,55,56,58,59,63,66,67,69-75,78-84,86-93,95,97,98]
		HCV	[61,65]
		HBV & HCV	[53,62,76]
		HBV & liver cancer	[54,57,77,96]
		Blood-borne viral infections	[64]
People with chronic HBV	2	HBV	[94,97]
Injecting drug users with HCV	1	HCV	[59]
General population and people with HBV	1	HBV	[52]

Most studies examined the views and experiences of South East Asian immigrants from China, Korea, Cambodia, and Vietnam in USA, Canada, or Australia; with only two focussing on other non-Asian ethnic groups- Turkish and Egyptian immigrants (see Table 6). The cross-sectional survey studies (Additional file 1: Table S2) used a variety of survey questionnaires derived from diverse theoretical models: seven were based on the constructs of Health Behaviour Framework [49,59,74-76,81,87]; one study [86] used Predisposing, Reinforcing and Enabling Constructs in Educational Diagnosis and Evaluation (PRECEDE) framework [100]; and six surveys used adapted questionnaires from published studies that used the Health Belief Model [52,53,60,67,69,94]. Other surveys (Additional file 1: Table S3) did not clearly indicate the theoretical frameworks that influenced their choice of survey questions and used different methodologies, and focussed only on HCV, or HBV and HCV together, with none focussing only on HBV [54,57,62,63,66,71-73,77,80,82,84,85,88-90,93,97-99].

**Table 6:** **Populations of focus**

**Populations**	**Number of studies**	**Sources**
Chinese	16	[53,55,56,58,59,63,70,72,73,84,88,89,92,93,96,97]
Vietnamese	13	[50,64,66,71,74-76,81,85-87,93,95]
Cambodian	6	[49,53,74,80,82,83]
Korean	4	[48,57,69,74]
Mixed Asians	7	[52,60,61,67,77-79,94]
Laotians	2	[53,98]
Hmong	4	[51,52,68,74]
Turkish	2	[90,91]
Egyptians	1	[65]
Mexican Americans	1	[62]

Qualitative studies (Additional file 1: Table S4) used various methods: combination of open-ended interviews and focus groups [51,58,95]; focus group [50,55,68,78,91]; semi-structured and in-depth interviews [56]; observational and in-depth interviews [65]; and ethnography [61]. A mixed methods study used both survey questionnaires based on Health Behaviour Framework and in-depth-interviews [69] (Additional file 1: Table S5).

Most of the surveys used a convenience sample (e.g. attendees of health clinics or community events). Even though, surveys were administered in the language of the participants, and many had good response rates, many relied on telephone surveys, which may have excluded participants who did not have telephones or take phone calls; and the reliance on self-reported data may have skewed some of the results. In addition, because of the diverse data collection methods in surveys, comparing data across studies as well as ethnic groups was only possible to a very limited extent.

The findings derived from the data were synthesised according to the explanatory model framework and presented in specific categories of: concepts of HBV and HCV; symptoms; causes; transmission; prevention; consequences; and treatment of HBV and HCV infections.

### The concepts of hepatitis B and C infections

In their descriptions of HBV infection, South East Asian participants (Vietnamese, Cambodians, Chinese and Koreans) appropriated biomedical information and used terms such as ‘liver sickness’ , ‘liver disease’ , and ‘liver cancer’ [50,51,56,58,78,95]. Cambodians referred to HBV infection as ‘blood disease’ , ‘bad blood’ , ‘contaminated blood’ , ‘unclean blood’ and ‘yellow skin’ [50]. Some Vietnamese participants [84] used the term, *benh viem gan* B, which translates to mean HBV. Participants from Hmong ethnic group who had HBV referred to it as “*mob ntshav daj”*, meaning “illness that makes your blood yellow” [69].

Two surveys [56,64] and three qualitative studies [50,68,95], reported on participants’ confusion about different types of viral hepatitis. For instance, a study in Australia found that some South East Asians with chronic HBV understood ‘viral hepatitis’ as existing at three levels - A, B, and C, with C being the worst. Some participants perceived linear and progressive stages of the same illness evolving from hepatitis A, to hepatitis B, and then to hepatitis C [50,95]; and some considered that this might progress further to immunodeficiency virus (HIV) [95]. In another study, however, respondents perceived hepatitis A as deadly, B as not very good, and C as okay; with some offering explanations using different anatomical locations of the infections: hepatitis A is on the skin only, while B reaches the liver [50].

### The symptoms of hepatitis B and C infections

Some of the Chinese, Vietnamese, and Korean participants associated HBV infection with symptoms such as ‘yellow skin’ , ‘yellow eyes’ , ‘yellow blood’ and ‘fatigue’ (‘looking tired’) [50,56,58,95]. In addition, Cambodians associated HBV infection with ‘tough liver’ , ‘swollen belly’ , and ‘dysfunctional liver’ [50]. Between 23% to 83% of participants knew of transmission risk by healthy looking subjects [49,53,59,60,64,67,69,76,81-83,85-99], and recognised that HBV infection can be asymptomatic.

Though inter-ethnic differences were found, whereby Cambodian participants in two studies had the least level of knowledge (23% and 43%) compared to other South East Asian groups, there are also intra-ethnic differences as evidenced in the way that different studies found both high and low levels of knowledge in Chinese participants.

### The causes of hepatitis B and C infections

The causes of HBV and HCV were attributed to many factors in various studies (Table 7 provides a detailed synthesis). Vietnamese, Chinese and Korean participants in some studies believed that a ‘weak liver’ causes HBV rather than vice versa. Various factors such as harmful foods [51,56], stressful lifestyle [78], hormones [51], poor nutrition [68], environmental pollutants [51], smoking [56], and alcohol [56] were perceived as causing weak liver, which in turn was seen to cause HBV. In one study, Vietnamese participants attributed the causes of weak liver to hard labour, starvation, political persecution, and physical deprivations that the Cambodians and Vietnamese experienced in prisons and camps during war [51].

**Table 7:** **Categorisation of the outcome data according to the explanatory model framework**

**Theme**	**Category**	**Subcategory**
The causes of HBV and HCV	Damaged liver/weak liver [50,55,67,77]	
	Genes and hormones [50,65,77,79]	
	Stress and negative emotions [50,51,55,67,77]	
	Supernatural beliefs & *kama*/fate [48-50,77,86]	
	Poor sanitation and hygiene [49,94]	
	Alcohol [61,67,77]	
	Smoking cigarettes [75]	
	Food/diet [49,66]	
	Physical exhaustion and fatigue [55,67]	
	Internal and external imbalance [49,50,68,77,79]	
	Fatigue [66]	
	Bacteria [49]	
HBV and HCV transmission factors	Sexual transmission [48-52,58,59,61-63,66,68-72,75,76,79-93,96-98]	
	Intravenous needles for drugs [52,60,61,64,66,68,76,81,96] and other drug injecting paraphernalia - spoons, swabs, water, tourniquets, and filters [60,61,64,76]	
	Through child birth or pregnancy [48,50-52,58,59,61,63,65,66,68-71,74-76,79,82-87,91-93,96,97]	
	Mosquito bites [61,64,65]	
	Poor hygiene/sanitation [49,77,79]	
	Contact with contaminated objects/materials [55,57,77,79]	
	Tattoos and body piercing [60,61,65,76]	
	Environment	Remoteness of a place [49]
		Poor conditions of living [49]
		Farming (livestock and poultry) [49]
	Contact with blood and other body fluids	Blood [52,57,59,66,79,96]
		Saliva [64]
		Cough and nasal discharge [49,69,74,75,80-83,87-89,98]
		Breast milk (breast feeding) [61]
		Premasticated food [80,81,87-89]
	Eating and drinking practices	Eating food prepared by an infected person [63,68,71,72,80-84,87-89]
		Sharing food & drinks with an infected person [67,70,79,97]
		Sharing eating utensils [48,59,61,63,65,67-69,71,75,79,83,84]
		Consumption of contaminated food [57,61,66,79,96]
		Consumption of raw food [59,79]
		Drinking contaminated water [61,67]
	Intact person-to-person contact	Hugging [55,61,65,71,92]
		Shaking hands [55,61,65,71,92]
		Holding hands [55,80,81,83,87,89]
		Kissing [77,79]
	Non-intact person-to-person contact	Bites or wounds [64]
		Open mouth ulcer [64]
	Sharing personal items	Sharing toothbrush [52,61,65,68,69,71,72,75,80-83,86-89,93,96]
		Sharing razors, blades and scalpels [52,57,60,61,63,65,68,81,83,84,87,96]
		Sharing earrings [50,79,81]
	Iatrogenic transmission	Sharing needles for injections [66,71,72,75,79,83,98]
		Medical equipment [93]
		Traditional healing practices – coin rubbing, cupping & moxibustion [61], acupuncture [61,87] and lancet therapy [87]
		Blood transfusion [50,52,57,61,62,65,77]
	Heredity	Genes [69,79]
Prevention of HBV and HCV	Vaccinations [51,52,54-57,59,62,65-67,70-72,77,78,84,90,91,94,96,98]	
	Screening [54,59,62,71,72,74,78,86,90]	
	Good hygiene practices [55,57,59,67,72,78,90]	
	Avoid contact with blood and other body fluids [61,78]	
	Avoid sharing drug injecting needles/paraphernalia [61]	
	Condoms [65]	
	Not taking illicit drugs [78]	
	Traditional medicines [49,55,58,67]	
	Regular hospital check-up [78]	
	Consumption of clean and properly cooked food [76,78,81,82,84,87,88,96]	
	Avoid infected needles [52]	
	Clean water [52,66,72,78,81,82,84,85,88,96]	
	Altering eating and drinking practices	Not sharing utensils and food [55,57,61,67,72,78,81,82,84,87,88,96]
		Not eating in restaurants [78]
	Adopting healthy lifestyles and practices	Good nutrition & balanced/healthy diet/healthy living [55,67,71,78,85]
		Regular exercise [55,67,77,78,87]
		Enough rest [55,67,77,87]
		Stress-free mind and positive attitude [55]
		Abstain from smoking [55,78]
		Abstain from drinking alcohol [55,78]
	Avoid contact with infected individuals	No kissing [55,67,78]
		No touching [55,67,78]
		No hugging [55,67,78]
		No contact with infected individuals [55,67,78]
		Avoid public spaces [78]
		Avoid public bus [78]
	Avoid sharing personal effects	Not sharing razors & shaving blades [78]
		Not sharing toothbrush [61,78]
		Not sharing soap [61]
Consequences of HBV and HCV infections	Chronic infection [48,52,58,59,62,63,70,74-76,80-82,84,86,88,91-93,98]	
	Depletion of energy from the body [86]	
	Depression [68]	
	Death [48,52,68,75,80,81,85,86,88,89,91,92,94,96,98]	Short life expectancy [52]
	Poor health [52]	High blood pressure [81,89]
		Stomach ulcer [81]
	Discrimination and stigma	Discrimination [52,64]
		Stigma [48,52,54,59,60,64-66,70,74,77,90,93,97]
		Avoidance [48,54,58,64,66,74,75,77,86]
		Isolation [52,54,94]
		Parental rejection [90]
		Fear for the future [48]
		Burdening the family [48,90]
		Shame [48,52,60,90]
	Loss of income	Loss of employment [52]
		Un-employability [48,52,90]
	Loss of social status	Not having children [52]
		Spoilt identity [60]
		Impediment to future marriage [90]
	Liver disease	Liver cancer [48,51,52,56,58,59,62,63,66,68-75,80,81,83-86,88,89,91,92,96-98]
		Liver cirrhosis [49,52,55-57,59,62,63,66,70,84,94,96,97] Adversely affect liver [49,55,57,94]
		Liver failure [52]
Treatments for HBV and HCV infections	Hospital treatment/medicine [61,67]	
	Good nutrition [67]	
	Lifestyle changes	Adequate rest and meditation [67]
		Physical Exercise [67]
		Avoid alcohol [67]
	Indigenous medicines	Acupuncture [49,50,61,67,71,73,78]
		Herbal remedies [49,50,55,67,78]

Lay explanations of cause(s) of HBV in different studies among South East Asians included poor sanitation and hygiene [50,95] and biological factors such as genes and hormones [50,70,78,80]. The cause of HBV was popularly attributed to stress and negative emotions [51,70,78,80], *karma* or fate [49-51,78,87], ‘imbalance’ in the body [50,51,56,78], and lifestyles factors such as fatigue [56,67,68]. Only Cambodian participants mostly attributed the cause of HBV to food [50], while Chinese, Vietnamese and Korean participants also mentioned alcohol [68,78]. The attribution of cause of HBV to smoking and bacteria were only made by the Vietnamese [76] and Cambodians [50] respectively.

### Transmission of viral hepatitis B and C infections

Immigrants attributed the transmission of HBV and HCV to many factors (Table 7). Korean, Chinese, and Vietnamese participants perceived HBV infection as contagious [55,56,58,78], and easily transmissible through multiple routes that are related to daily activities such as food or physical contact with infected people or objects [56,78,95].

The various surveys showed that 19% to 80% of South East Asian participants knew that HBV could be sexually transmitted [49,52,53,59,60,64,67,69-73,76,77,80-99]. There were both inter- and intra-ethnic as well as regional variations in the level of this knowledge. For instance, only 1 in 5 (19%) participants among Koreans correctly identified sexual behaviour as a risk factor [67], which is at the lower range of estimates reported by similar studies of Chinese and Vietnamese immigrant populations in the United States and Canada [64,73,85,88]. In terms of intra-ethnic and regional differences, awareness of sexual transmission risk was found to be as low as 23% in Philadelphia, to as high as 71% in Seattle, among Vietnamese immigrants [67,72,82,87,88,94], compared to a range of 40% to 80% among Chinese in the Netherlands, USA, and Canada [59,60,64,71,72,85,93,94,97,98].

Factors perceived to increase the risk of horizontal transmission in surveys were: contact with contaminated blood or other body fluids from infected person (reported by 61% to 90% of participants) [53,60,67,80,97]; premasticated food (reported by 63% to 82% of participants) [53,69,81,82,88-90]; contaminated drug injecting needles and syringes (reported by 59% to 86% of participants ) [53,67,69,82,97]; sharing of toothbrush (reported by 33% to 86% of participants) [49,52,53,67,69,72,73,76,81-84,87-90,94,97]; and sharing of razors (reported by 55% to 80% of participants) [53,64,69,82,84,85,88,97]. The level of knowledge of transmission through sharing of toothbrush was lowest among the Samoa and Chamorro [80] and Hmong [52], with evidence of intra-ethnic variation among the Vietnamese ranging from 42% to 77% [67,72,76], probably due to different sample sizes.

South East Asians commonly attributed transmission of HBV infection to factors such as contaminated and uncooked or poorly cooked food [53,58,60,67,69,80,97] and communal sharing of food and drinks [50,51,56,58,78,95]. Surveys showed that between 21% and 71% of participants believed that HBV infection could be transmitted through eating of food prepared by an infected person [53,64,69,72,73,81-85,88-90], and between 26% to 48% of the Chinese participants [71,80,98] knew that HBV infection could be transmitted through sharing of food and drinks. Between 15% to 89% of participants knew that HBV infection could be spread through sharing utensils [49,60,64,69,70,72,76,80,84,85]. The attribution of transmission of HBV through food and sharing of utensils is particularly influenced by the cultural practices around food preparation, and eating and drinking habits among the South East Asians [51,58,68,95]. In a number of studies, 58% to 76% of participants also believed that HBV infection could be transmitted through holding hands [69,81,82,84,88-90].

Between 34% and 91% of participants in surveys knew of the vertical transmission of HBV infection through child birth [49,52,59,60,64,67,69-71,74-76,80,83-88,92-94,97,98]. This knowledge was low in some studies with Korean participants (34%) [70] and Samoa and Chamorro participants (38%) [80], but tended to be high among Chinese participants (range 76% to 91%) [59,60,71,85,94]. Nonetheless, the results from different studies indicate intra-ethnic variations, as the level of knowledge of this risk factor is low among Koreans in Rocky Mountain [70], but high among Koreans in Los Angeles [49], Vietnamese Americans (59-85%) [67,72,75,86-88], and Vietnamese Australians (67%) [94]. In contrast, a single study of Chinese and Vietnamese Australians showed no significant ethnic differences in the level of knowledge of this mode of transmission [94]..

There is evidence of knowledge of transmission of HBV through contaminated (therapeutic) injection practices [51,58,63,67,72,73,76,78,80,84,88,94,99]. The proportion of survey participants who knew of this risk factor varied between studies from 18% to 92% [67,72,73,76,80,84,99]. The level of knowledge of this risk factor was low among the Laotians [99], but high (92%) among the Cambodians [84]. There was evidence of both intra- and inter-ethnic variations among the Vietnamese ([67,72,76]; between 50% to 85%) and Chinese ([73,97]; between 52% to 85%).

Some immigrants knew that HCV infection could be transmitted vertically through child birth and breast feeding [62,66], or horizontally through sex [62,63], blood transfusion [62,63,66], intact skin contact (such as hugging and shaking hands [62,66]), non-intact skin contact (such as bites or wounds [65] and open mouth ulcer [65]), tattooing and body piercing [61,62,66,77], razors and shaving blades [61,62,66], sharing of contaminated drug injecting paraphernalia [61,62,65,77], sharing toothbrush [62,66], and traditional healing practices (such coin rubbing, cupping, moxibustion, and acupuncture) [62]. Some immigrants also believed that HCV could be transmitted through ingestion of contaminated food [62], water [62,68], sharing of utensils [62,66] and saliva [65].

Some studies suggests that the framing of the transmission of HBV infection within a broader issue of sexually transmitted disease [49,55,65,78,91,95], and injecting drug use [61,65,95] contributed to its association with shame and stigma.

### Prevention of viral hepatitis

Immigrants explained the prevention of HBV and HCV in various ways (Table 7). Survey studies showed that between 54% and 96% of participants knew that HBV could be prevented through vaccination [49,52,53,57-60,67,71,72,92,97-99], with no significant difference among the ethnic groups, or wide intra-ethnic variation between studies. However, despite the relatively high level of awareness of vaccination for HBV, some of these studies indicate that the levels of self-reported vaccination rates in the Asian-American populations were low [56,58,72,81,85,87,88,90,97].

Studies that explored immigrants’ experiences with vaccination have found mixed results among and between ethnic groups. While some Chinese [55,58,91], Koreans [55,58,91], and Turkish-Dutch [55,58,91] participants’ attitudes towards vaccination were generally positive and many were receptive to being vaccinated, others (among South East Asian groups) were confused and uncertain about the purpose of vaccination, its cost, efficacy and benefits (including side-effects), and the number and frequency of shots needed [50,55,56,58,68,95]. The lack of provision of adequate information on HBV vaccines and process of vaccination by healthcare professionals appeared to add to the confusion among Chinese participants [56]. As such awareness about the knowledge of the existence of vaccines for HBV did not translate into acceptance that vaccination should be used as the primary means of preventing HBV infection among this Chinese group [56]. However, some south East Asian participants were found to perceive vaccination positively following active recommendations by doctors and healthcare providers [59,61,64,71,72,76,87,90,97], family or friends or if it was compulsory for employment or school admission [58,67,90]. In addition, some participants from Chinese, Pacific Islanders, and Vietnamese immigrants indicated that targeting the whole family for intervention might positively influence uptake of screening and vaccination [79,80,87].

In relation to attitudes to screening, surveys showed that 32% to 94% of South East Asian participants (Chinese, Vietnamese, Hmong and Koreans) were aware of the availability of screening for hepatitis B infection [60,72,73,75]. There were high intra- and inter-ethnic and regional variations in levels of knowledge of screening as a means of preventing hepatitis B infection. For instance, knowledge of screening was low (32%) among the Vietnamese in USA [72,75], but higher among Koreans (94%) in the USA [60,72,75] and Chinese in Canada [71,73] . It is not clear why the Vietnamese in Philadelphia and New Jersey had poor knowledge of screening compared to other communities, though this is also reflected in low levels of rates of screening among other Vietnamese immigrants (around 6% to 8%) [72,82,96], suggesting that better knowledge might influence uptake of screening.

Though some immigrants expressed general motivation to, or actively sought, screening [55,56,87,91,95,97,99] to prevent liver disease [63,75,87], some with experience of screening held mixed views. To some Cambodian, Chinese, and Vietnamese participants, screening caused shock and anxiety due to lack of provision of adequate information at pre-screening and post-screening stages by healthcare professionals, and in some cases people were routinely tested without explicit consent [95]. That created considerable confusion and fear as people were not only unaware of the likely impact of the infection on their health. These experiences might also influence future health-seeking behaviours of immigrants.

In addition, lack of provision of adequate health information led to missed opportunities for provisions of knowledge and information about viral infections which could have provided avenues for health promotion and behaviour change [95]. The evidence also suggests that a better knowledge of screening process and procedures, and comprehension of test results might positively influence engagement with screening and vaccination [49,55,56,79,95].

Some evidence also suggests that compulsory screening [91], and the recommendation by healthcare professionals might be positively associated with uptake of screening and vaccination [59,61,64,71,72,76,90,97]. The perceived advantages of ‘targeting’ might partly be due to its resonance with some of the migrants’ experiences with active public health interventions, including compulsory screening and vaccinations in their countries of origin [58].

### The consequences of hepatitis B & C infection

Participants in qualitative studies and surveys considered that HBV and HCV infections led to both health-related problems (liver cirrhosis, liver cancer, chronic infection and death) and socio-economic ones (discrimination and stigma, loss of income, and loss of social status) (Table 7). The awareness of consequences of HBV infection included liver cirrhosis (reported by 40% to 98% of participants) [57,60,64,67,71,85,97,98]; liver cancer (reported by 25% to 92% of survey participants) [53,69,75,76,81-83,85-87,89,90,92-94,97-99]; chronic infection (reported by 24% to 75% of survey participants) [49,53,59,60,64,69,71,75,76,81-83,85,87,89,90,92-94,99]; and death (reported by 36% to 93% of survey participants) [49,53,69,76,81,82,86,87,89,90,92,93,97,99]. Findings from qualitative studies showed that some South East Asian participants (Cambodian, Hmong, Korean, and Chinese) were aware that HBV infection could cause liver cancer and/or liver failure [50,53,56,58,63,69,95].

In relation to stigma and shame, between 36% to 70% of South East Asian participants in surveys believed that people with chronic HBV infection are sometimes avoided by others [49,55,59,65,67,75,76,78,87]. This avoidance was perceived to occur both at the family and community levels [53,55,78,95,98]. Some Turkish-Dutch [91] participants perceived an infection with HBV as an impediment to future marriage and employment. Some South East Asians also associated HBV infection with impediment to getting employment [49,53,91]. Shame and stigma might negatively influence uptake of screening; and those who test positive might not disclose their test results [95,98], and might continue to spread infections or develop fatal sequelae.

### Treatments for hepatitis B & C infections

In nine surveys, between 44% to 96% of the participants knew about treatments for HBV infection [52,60,71,75,83,87,94,98,99]. One survey also reported knowledge of lack of effective treatment for HCV infection [66].

Some of the studies included in this review identified varying levels of beliefs or perceptions (correct or otherwise) of the nature and range of treatments for HBV and HCV infections including internal and external restoration of balance through lifestyle changes and good nutrition [68]; indigenous medicines [50,51,68,79]; and hospital medicine [50,68] (Table 7). Evidence indicates that South East Asian (Cambodian, Vietnamese, Chinese, and Korean) immigrants residing in North America, mainly base their treatments for HBV on lifestyle changes, diet, and restoration of internal and external balance in the body [50,51,68,79]. Even if people use hospital (Western) medicine to treat symptoms of HBV infection [50,68], they also use traditional medicine because of its affordability [68], or as a last resort when the former fails to effectively *cure* HBV infection [50,51,68].

The only study that investigated Indo-Chinese (Cambodian, Lao, and Vietnamese), immigrants’ experiences with treatments identified key aspects of service experience including long waiting lists, problems with obtaining a doctor’s referral, and difficulties with making and keeping appointments [61]. Even though the study’s sample comprised only injecting drug users, the findings could have implications for interventions across immigrant groups.

### The correlates of knowledge of HBV and HCV infections

Variation in the levels of various forms of knowledge of HBV and HCV among and between ethnic groups is influenced by several factors. Some of the surveys examined some of these correlates of knowledge. Better level of knowledge of HBV was related to socio-economic and demographic factors such as higher levels of education [56-58,70,75,84,87-90,93,97,98], income [57,75,88-90,98], and English proficiency [56,58,67,70,75,77,81,84,90]. Even though there is evidence from surveys [60,75,79,84,98] and qualitative studies [50,78] that indicate a correlation between younger age and better knowledge, this was not significant in some surveys [82,83,97]. Similarly, inconclusive evidence implies that better knowledge of HCV is correlated with higher levels of education [66], younger age [66,69], house ownership [57,82,88-90], employment [66,77], having had vaccination for HBV [77] or having sought preventive care [69]. In addition, being aware of HBV [57,88-90], and having received health education through mass media [57,88-90] was correlated with better knowledge.

Personal experiences with HBV infection [56,58,60,91,95], having a family member with HBV or liver cancer [50,60,67,81,97], screening [64,67,71,73,76,94,97], and vaccination [67,94] were associated with better knowledge; though in one study, individuals who had a personal or family history of HBV or liver cancer were more likely to have been screened, but they did not have better knowledge of HBV [97]. Evidence from one qualitative study among those with chronic HBV, also suggests that people who had access to specialist care [98] had better knowledge about the infection.

In some studies, being born in a host country (USA) [69,84] was correlated with better knowledge of HBV. Even though younger age at immigration might be positively correlated with better knowledge of hepatitis B [84], the longer immigrants stay in host country, the more they associate HBV and HCV with stigma [95]. However, given the small number of studies in this area, these conclusions are preliminary.

There was variation among ethnic groups (e.g. Koreans, Cambodians, Vietnamese, and Chinese) on the level, and type, of knowledge of HBV, including attitudes towards, and understandings of, vaccination, screening, treatment, reception of medical advice, and stigma [57,68,75,78,82,85,94,97]. For instance, the Chinese and Korean groups discussed vaccination as a preventive measure more so than the Vietnamese groups [68]. Compared to Chinese and Vietnamese groups, Koreans did not understand the significance of screening prior to vaccination or taking up vaccination when one is healthy [68]. More often than other groups, Chinese participants mentioned poor hygiene, and Vietnamese groups mentioned drinking alcohol as a cause of HBV infection [68]. There were also differences in the perceptions of stigma by ethnic group: only 30% of Vietnamese respondents believed that people with HBV infection were to be avoided compared to 70% of Cambodian respondents [75].

More Vietnamese than Chinese knew that hepatitis B infection could be asymptomatic or could lead to liver cancer, whereas more Chinese than Vietnamese identified the disease as life-long and that treatment were available [94]. In another study, Koreans were more likely to affirm that HBV infection causes liver cancer than the Hmong, and perceived susceptibility was highest among Korean Americans and lowest among Hmong [75]. The Turkish-Dutch participants were the least aware that HBV infection could cause death [92], followed by Chinese in the Netherlands [93], while Chinese Americans have consistently reported high level of awareness. In contrast, evidence implies that approximately 1 in 3 Vietnamese respondents were not aware that HBV infection could cause liver cancer [67,72,75,76,82,87,94]. In addition, the knowledge of this aspect of HBV infection in the Australian Vietnamese sample was comparatively lower than that reported in studies among Chinese in North America [64,73,85]. The studies in our sample indicated that gender might influence the level of knowledge, with more women likely to believe that hepatitis B infection could be cured. One of the possible explanations for the variation of level of knowledge of treatability of hepatitis infection among the Cambodians [81], Laotians [99], and Chinese [71,89,90] compared to Vietnamese [76,82] could be gender as Cambodian [81] and Chinese samples [89,90] were either all female or made up of greater proportion of females [71,99]. One in six Chinese women [89,90] were aware that HBV could not be cured compared to a very low awareness of this among studies of male or mixed gender participants. It is not clear what impact the knowledge of either curability or incurability of HBV and HCV could differentially have on the men’s *vis a vis* women’s uptake of screening, vaccination, and treatment. Other studies found that gender did not have an influence on the level of knowledge of HBV [64,67,84,88,94].

Synthesised evidence suggests that other aspects of knowledge of HBV infection influence the understanding of the consequences of the infection. For instance, it seems that among the Chinese and Vietnamese respondents who knew that HBV infection can be asymptomatic had a better knowledge of HBV [95] than those participants who thought otherwise [64,85,86,90]. This in turn is likely to encourage people to seek screening to determine if they are infected rather than relying on experience of symptoms. Conversely, the belief that HBV infection is a transient infection [71,98] would lead to it not being taken seriously.

## Discussion

### Summary of findings

The evidence in the systematic narrative review has mostly emerged from studies of South East Asians in the USA, Canada, and (to a lesser extent) Australia. We found very little research on immigrants from other areas with high prevalence of HBV and HCV infections, such as Central Asian republics, South Asia, Africa, Middle East, and Eastern Europe. Most studies in our sample investigated knowledge of HBV infection. Most studies identified were surveys, with a few qualitative studies, generally confirming some of the quantitative findings. Different surveys showed striking differences in levels of awareness and knowledge of all aspects of HBV and HCV infections from the nature of these infections, and transmission factors, to how to prevent and treat them in both acute and chronic phases. Some of these differences, evidenced among and between ethnic groups, may be explained by socio-economic and demographic factors, country of origin (viral infections is highly stigmatised and people are discriminated against in places like China), and experiences with migration (some countries require migrants to be screened before immigration).

The evidence indicates that many, though not all, immigrants lack adequate knowledge of the aetiology, symptoms, transmission risk factors, prevention strategies, consequences and treatment of HBV and HCV infections. The lack of adequate knowledge is particularly evidenced in: (a) the disconnect between having heard of HBV infection or liver cancer and understanding of the health implications of chronic HBV and HCV infections; (b) confusion about the difference between various types of viral hepatitis; (c) the low level of knowledge of main transmission risk factors, especially sexual contact (horizontal transmission) and child birth (vertical transmission), and the incorrect attribution of cause and transmission principally to lifestyle activities and cultural practices around food; and (d) poor level of knowledge of chronicity of HBV infection. It is apparent that immigrants remain vulnerable to the spread of HBV and HCV infections within their ethnic communities; and some are dying prematurely from these infections, partly because of inadequate level of knowledge.

The published evidence indicates that many, though not all, South East Asian immigrant groups have inadequate or confused knowledge of HBV and HCV infections, and that this could affect their willingness to participate in screening and treatment programmes. For example, folk models of these conditions commonly attribute them to damaged or weak liver, poor hygiene and sanitation, and food-related factors. Transmission of HBV and HCV infections was often attributed by immigrants to sanitation, contaminated food, and cultural practices around the communal sharing of food and utensils and HBV infection was not widely perceived to be a sexually transmitted infection.

Another component of the ‘lay epidemiology’ of HBV infection in some immigrant groups is social factors such as incarceration, deprivation, hard work, stressful lifestyle, and physical exhaustion; these perceptions may reflect an *association* of the condition in some settings with social determinants of health (in particular, poverty and overcrowding), but the assumed *causal* link may interfere with health education about transmission of a blood borne virus. In some studies, immigrants had appropriated biomedical knowledge including medical terms and clinical markers to make sense of the infections. However, such (apparent) knowledge often masked a more general confusion about the nature and natural history of the conditions, partly because of the asymptomatic latent (but still highly contagious) stage was under-recognised, and because the symptoms associated with HBV and HCV infections such as abdominal pain, dark urine, fever, joint pain, nausea, fatigue, and jaundice are associated with many infectious diseases in the developing word.

### Strengths and limitations of this study

A particular strength of this review lies in its synthesis of findings from qualitative and quantitative studies. As such the review has provided rich evidence on the nature and type of knowledge of HBV and HCV infections as well as quantitative data on levels of knowledge. Even though we employed a Mixed Methods Appraisal Tool (MMAT) for methodological quality assessment of studies, we included all the publications, which added a layer of depth in our synthesis. Due to significant heterogeneity in methodological approaches and findings of quantitative studies, we did not undertake a meta-analysis. In addition, this has compromised the scope for comparison of results from different studies across regions, countries, as well as among and between immigrant ethnic groups. Specifically, we did not test for acculturation related factors that might associate with health literacy, nor were we able to make country comparisons, for example, of Chinese people in the US, Australia, or Canada.

Since we included only studies published in peer-reviewed journals in English, it is possible that we excluded some important publications, especially in grey literature such as theses and reports, but given the dearth of literature this is unlikely given out focus on ethnic groups and immigrants.

## Conclusions

Many immigrants have inadequate knowledge of HBV and HCV infections. These findings have implications for public health interventions aimed at stemming the rise in prevalence of HBV and HCV but also that of liver cancer through screening and treatment. Despite the differences in levels knowledge and types of explanatory models among different ethnic groups, the public health interventions indicated would be similar, just as the drivers of health-seeking behaviour. Using an adapted PEN-3 model [46-48], we have developed a guide (including strategies and approaches) that can be used by service providers targeting immigrants (Additional file 1: Table S6). We have further demonstrated how the model could be used to influence specific ‘negative’ knowledge, attitudes, and beliefs while also reinforcing the ‘positive’ or ‘neutral’ ones, most of which exist across the ethnic groups.

This review has identified several gaps in the current evidence of knowledge of HBV and HCV infections among immigrants. The overarching aim of further research would be to identify knowledge related barriers, and develop interventions, strategies, and approaches that might positively influence migrants’ uptake of screening, vaccination, and clinical management of chronic HBV and HCV, and prevent the rising rates of hepatocellular carcinomas, and liver cancer. In addition, further research is needed on: migrants’ experiences with chronic HBV and HCV; immigrants’ experiences with interventions (education, screening, vaccination, and treatment); immigrants’ knowledge of HCV; and knowledge of HBV and HCV infections among diverse range of migrant groups in different migrant-receiving countries. In addition, studies are needed to determine factors that influence the nature, types and level of knowledge that immigrants hold, and uptake of screening, vaccination and treatment (including what immigrants think could be appropriate interventions). The findings challenge public health institutes to develop local needs assessments and cross-national projects.

## Additional file

Additional file 1: Table S1. Reviewed studies. **Table S2.** A Summary of hepatitis B knowledge. **Table S3.** Other quantitative / survey studies: A summary of hepatitis B and/or C knowledge. **Table S4.** Qualitative studies: A summary of hepatitis B and/or C knowledge. **Table S5.** Mixed Methods studies: A summary of hepatitis B knowledge. **Table S6.** Translation and application of an adapted PEN-3 model of analyses.
