# Supplementary material for: Illness perceptions and explanatory models of viral hepatitis B & C among immigrants and refugees: a narrative systematic review
Source: BMC Public Health. 2015 Feb 15;15:151. doi: 10.1186/s12889-015-1476-0 (PMC4336715; doi:10.1186/s12889-015-1476-0)
Supplement: Additional file 1: Table S1. — Reviewed studies. Table S2. A Summary of hepatitis B knowledge. Table S3. Other quantitative / survey studies: A summary of hepatitis B and/or C knowledge. Table S4. Qualitative studies: A summary of hepatitis B and/or C knowledge. Table S5. Mixed Methods studies: A summary of hepatitis B knowledge. Table S6. Translation and application of an adapted PEN-3 model of analyses. [file 12889_2015_1476_MOESM1_ESM.docx]

*Supplementary Table 1: Reviewed studies*

| Bastani, R., Glenn, B. A., Maxwell, A. E., & Jo, A. M. (2007). Hepatitis B testing for liver cancer control among Korean Americans. *Ethnicity and Disease*, 17(2):365-373.  Burke, N. J., Jackson, J. C., Thai, H. C., Stackhouse, F., Nguyen, T., Chen, A., & Taylor, V. M. (2004). ‘Honoring tradition, accepting new ways’: development of a hepatitis B control intervention for Vietnamese immigrants. *Ethnicity & Health*, *9*(2):153-169.  Burke, N. J., Do, H. H., Talbot, J., Sos, C., Svy, D., & Taylor, V. M. (2011). *Chumnguh Thleum*: Understanding Liver Illness and Hepatitis B Among Cambodian Immigrants. *Journal of Community Health*, *36*(1): 27-34.  Butler, L. M., Mills, P. K., Yang, R. C., & Chen Jr, M. S. (2005). Hepatitis B knowledge and vaccination levels in California Hmong youth: Implications for liver cancer prevention strategies. *Asian Pacific Journal of Cancer Prevention*, *6*(3): 401-403.    Carabez, R. M., Swanner, J. A., Yoo, G. J., & Ho, M. (2014). Knowledge and Fears Among Asian Americans Chronically Infected with Hepatitis B. *Journal of Cancer Education*, 29 (3): 522-528.    Caruana, S. R., Kelly, H. A., Silva, S. L. D., Chea, L., Nuon, S., Saykao, P., Bak, N. & Biggs, B. A. (2005). Knowledge about hepatitis and previous exposure to hepatitis viruses in immigrants and refugees from the Mekong region. *Australian and New Zealand Journal of Public health*, 29(1): 64-68.  Chang ET, Nguyen BH, & So SK. (2008). Attitudes toward hepatitis B and liver cancer prevention among Chinese Americans in the San Francisco Bay Area, California. *Asian Pacific Journal of Cancer Prevention,* 9(4):605-13.  Chen, H., Tu, S. P., Teh, C. Z., Yip, M. P., Choe, J. H., Hislop, T. G., Taylor, V. M., & Thompson, B. (2006). Lay beliefs about hepatitis among North American Chinese: implications for hepatitis prevention. *Journal of Community Health*, 31(2): 94-112.  Cheung, J., Lee, T. K., Teh, C. Z., Wang, C. Y., Kwan, W. C., & Yoshida, E. M. (2005). Cross-sectional study of hepatitis B awareness among Chinese and Southeast Asian Canadians in the Vancouver-Richmond community. *Canadian Journal of Gastroenterology = Journal Canadian de Gastroenterologie*, *19*(4):245-249.  Choe, J. H., Chan, N., Do, H. H., Woodall, E., Lim, E., & Taylor, V. M. (2005). Hepatitis B and liver cancer beliefs among Korean immigrants in Western Washington: Report of a qualitative study. *Cancer*, 104(S12):2955-2958.  Coronado GD, Taylor VM, Tu SP, [Yasui Y](http://www.ncbi.nlm.nih.gov/pubmed?term=Yasui%20Y%5BAuthor%5D&cauthor=true&cauthor_uid=17940869), Acorda E, Woodall E, Yip MP, Li L, [Hislop TG](http://www.ncbi.nlm.nih.gov/pubmed?term=Hislop%20TG%5BAuthor%5D&cauthor=true&cauthor_uid=17940869). 2007. Correlates of hepatitis B testing among Chinese Americans. *Journal of Community Health,* 32(6):379-90.  Cotler, S. J., Cotler, S., Xie, H., Luc, B. J., Layden, T. J. and Wong, S. S. (2012). Characterizing hepatitis B stigma in Chinese immigrants. *Journal of Viral Hepatitis*, 19:147-152.  Coupland, H., & Maher, L. (2010). Notions of Injecting Drug Users' Candidacy for Hepatitis C Treatment: Conflicting Provider, Patient, and Public Health Perspectives. *Contemporary Drug Problems*, *37*:549-573.  Dev, A., Sundararajan, V., & Sievert, W. (2004). Ethnic and cultural determinants influence risk assessment for hepatitis C acquisition. *Journal of Gastroenterology and Hepatology*, *19*(7):792-798.  Flores, Y. N., Lang, C. M., Salmerón, J., & Bastani, R. (2012). Risk factors for liver disease and associated knowledge and practices among Mexican adults in the US and Mexico. *Journal of Community Health*, *37*(2):403-411.  Hislop, T. G., Teh, C., Low, A., Li, L., Tu, S. P., Yasui, Y., & Taylor, V. M. (2007). Hepatitis B knowledge, testing and vaccination levels in Chinese immigrants to British Columbia, Canada. *Canadian Journal of Public Health. 98*(2):125-129.  Ho, H. T., Ho, H. T., Maher, L., Ho, H. T., & Maher, L. (2008). Có vay có tr (What goes around comes around): culture, risk and vulnerability to blood-borne viruses among ethnic Vietnamese injecting drug users. *Drug and Alcohol Review*, *27*(4):420-428.  Horwitz, R., Brener, L., Treloar, C., & Sabri, W. E. (2010). Hepatitis C is an Australian Migrant Community: Knowledge of and Attitudes towards Transmission and Infection. *Contemporary Drug Problems*, 37: 659-683.  Hwang, J. P., Huang, C. H., & Jenny, K. Y. (2008). Knowledge about hepatitis B and predictors of hepatitis B vaccination among Vietnamese American college students. *Journal of American College Health*, *56*(4):377-382.  Hwang, [J.P.,](http://link.springer.com/search?facet-author=%22Jessica+P.+Hwang%22)  Roundtree, A.K. & [Suarez-Almazor](http://link.springer.com/search?facet-author=%22Maria+E.+Suarez-Almazor%22), M.E. (2012). Attitudes Toward Hepatitis B Virus among Vietnamese, Chinese and Korean Americans in the Houston Area, Texas. *Journal of Community Health,* 37 (5):1091-1100.  Kue, J., & Thorburn, S. (2013). Hepatitis B Knowledge, Screening, and Vaccination among Hmong Americans. *Journal of health care for the poor and underserved*, 24(2): 566-578.  Lee, H-O., Lee O-J., Kim, S, Hontz, I., & Warner, A. (2007). Differences in Knowledge of Hepatitis B Among Korean Immigrants in Two Cities in the Rocky Mountain Region.  *Asian Nursing Research,* 1(3):165–175.  Li, D., Tang, T., Patterson, M., Ho, M., Heathcote, J., & Shah, H. (2012). The impact of hepatitis B knowledge and stigma on screening in Canadian Chinese persons. *Canadian Journal of Gastroenterology,* 26(9), 597-602.  Ma, G. X., Shive, S. S., Toubbeh, J., Wu, D., & Wang, P. (2006). Risk Perceptions, Barriers, and Self-Efficacy of Hepatitis B Screening and Vaccination among Chinese Immigrants. *International Electronic Journal of Health Education*, 9, 141-153.  Ma, G. X., Shive, S. E., Fang, C. Y., Feng, Z., Parameswaran, L., Pham, A., & Khanh, C. (2007). Knowledge, attitudes, and behaviors of hepatitis B screening and vaccination and liver cancer risks among Vietnamese Americans. *Journal of Health Care for the Poor and Underserved*, *18*(1): 62-73.  Ma, G. X., Shive, S. E., Toubbeh, J. I., Tan, Y., & Wu, D. (2008). Knowledge, attitudes, and behaviors of Chinese hepatitis B screening and vaccination. *American Journal of Health Behavior*, *32*(2):178-187.  Maxwell, A. E., Stewart, S. L., Glenn, B. A., Wong, W. K., Yasui, Y., Chang, L.C., Taylor, V.M., Nguyen, T.T., Chen Jr, M.S. & Bastani, R. (2012). Theoretically Informed Correlates of Hepatitis B Knowledge among Four Asian Groups: The Health Behavior Framework. *Asian Pacific Journal of Cancer Prevention*, 13:1687-1692.  Nguyen, Tung T., Stephen J. McPhee, Susan Stewart, Ginny Gildengorin, Lena Zhang, Ching Wong, Annette E. Maxwell, Roshan Bastani, Vicky M. Taylor, and Moon S. Chen. (2010). Factors associated with hepatitis B testing among Vietnamese Americans. *Journal of General Internal Medicine,* 25 (7): 694-700.  O'Connor, C. C., Shaw, M., Wen, L. M., & Quine, S. (2008). Low knowledge and high infection rates of hepatitis in Vietnamese men in Sydney. *Sexual Health*, *5*(3), 299-302.  Philbin, M. M., Erby, L. A., Lee, S., & Juon, H. S. (2012). Hepatitis B and Liver Cancer Among Three Asian American Sub-Groups: A Focus Group Inquiry. *Journal of Immigrant and Minority Health*, 14: 858-868.  Shiau, R., Bove, F., Henne, J., Zola, J., Fang, T., & Fernyak, S. (2012). Using Survey Results Regarding Hepatitis B Knowledge, Community Awareness and Testing Behavior Among Asians to Improve the San Francisco Hep B Free Campaign. *Journal of Community Health*, *37*(2):350-364.  Takahashi, L. M., Kim, A. J., Sablan-Santos, L., Quitugua, L. F., Aromin, J., Lepule, J., Maguadog, T., Perez, R., Young, L. & Young, S. (2011). Hepatitis B Among Pacific Islanders in Southern California: How is Health Information Associated with Screening and Vaccination? *Journal of Community Health*, *36*(1):47-55.  Taylor, V. M., Jackson, J. C., Pineda, M., Pham, P., Fischer, M., & Yasui, Y. (2000). Hepatitis B knowledge among Vietnamese immigrants: implications for prevention of hepatocellular carcinoma. *Journal of Cancer Education*, 15(1):51-55.  Taylor, V. M., Jackson, J. C., Chan, N., Kuniyuki, A., & Yasui, Y. (2002). Hepatitis B knowledge and practices among Cambodian American women in Seattle, Washington. *Journal of Community Health*, 27(3):151-163.  Taylor, V. M., Yasui, Y., Burke, N., Nguyen, T., Chen, A., Acorda, E., Choe, J. H., & Jackson, J. C. (2004). Hepatitis B testing among Vietnamese American men. *Cancer Detection and Prevention*, 28(3):170-177.  Taylor, V. M., Choe, J. H., Yasui, Y., Li, L., Burke, N., & Jackson, J. C. (2005). Hepatitis B awareness, testing, and knowledge among Vietnamese American men and women. *Journal of Community Health*, 30(6):477-490.  Taylor, V. M., Yasui, Y., Burke, N., Choe, J. H., Acorda, E., & Jackson, J. C. (2005). Hepatitis B knowledge and testing among Vietnamese-American women. *Ethnicity and Disease*, *15*(4):761-767.  Taylor, V. M., Tu, S. P., Woodall, E., Acorda, E., Chen, H., Choe, J., Li, L., Yasui, Y. & Hislop, T. G. (2006). Hepatitis B knowledge and practices among Chinese immigrants to the United States. *Asian Pacific Journal of Cancer Prevention*, 7(2), 313-317.  [Taylor, Victoria M](http://sharedresources.fhcrc.org/publications/search/author/2026); [Seng, Paularita](http://sharedresources.fhcrc.org/publications/search/author/3499); Acorda, Elizabeth; [Sawn, Lyvan](http://sharedresources.fhcrc.org/publications/search/author/3500); [Li, Lin](http://sharedresources.fhcrc.org/publications/search/author/1965) (2009). Hepatitis B Knowledge and Practices Among Cambodian Immigrants. *Journal of Cancer Education,* 24(2):100-104.  Taylor, V.M., Talbot, J., Do, .H.H., Liu, Q., Yasui, Y., Jackson, J.C., Bastani, R. (2011). Hepatitis B Knowledge and Practices among Cambodian Americans. *Asian-Pacific Journal of Cancer Prevention*, 12(4):957-61.  Thompson, M. J., Taylor, V. M., Jackson, J. C., Yasui, Y., Kuniyuki, A., Shin‐Ping, T. U., & Hislop, T. G. (2002). Hepatitis B knowledge and practices among Chinese American women in Seattle, Washington. *Journal of Cancer Education*, *17*(4):222-226.  Thompson, MJ, Taylor VM, [Yasui Y](http://www.ncbi.nlm.nih.gov/pubmed?term=Yasui%20Y%5BAuthor%5D&cauthor=true&cauthor_uid=12873087), [Hislop TG](http://www.ncbi.nlm.nih.gov/pubmed?term=Hislop%20TG%5BAuthor%5D&cauthor=true&cauthor_uid=12873087), [Jackson JC](http://www.ncbi.nlm.nih.gov/pubmed?term=Jackson%20JC%5BAuthor%5D&cauthor=true&cauthor_uid=12873087), [Kuniyuki A](http://www.ncbi.nlm.nih.gov/pubmed?term=Kuniyuki%20A%5BAuthor%5D&cauthor=true&cauthor_uid=12873087), [Teh C](http://www.ncbi.nlm.nih.gov/pubmed?term=Teh%20C%5BAuthor%5D&cauthor=true&cauthor_uid=12873087). 2003. Hepatitis B knowledge and practices among Chinese Canadian women in Vancouver, British Columbia. *Canadian Journal of Public Health,* 94(4):281-6.  van der Veen, Y. J., De Zwart, O., Voeten, H. A., Mackenbach, J. P., & Richardus, J. H. (2009). Hepatitis B screening in the Turkish-Dutch population in Rotterdam, the Netherlands; qualitative assessment of socio-cultural determinants. *BMC Public Health*, 9(1):328.  van der Veen, Y. J., Voeten, H. A., de Zwart, O., & Richardus, J. H. (2010). Awareness, knowledge and self-reported test rates regarding Hepatitis B in Turkish-Dutch: a survey. *BMC Public Health*, 10(1):512.  Veldhuijzen, I. K., Wolter, R., Rijckborst, V., Mostert, M., Voeten, H. A., Cheung, Y., Boucher, C.A, Reijnders, J.G., de Zwart, O. & Janssen, H. L. (2012). Identification and treatment of chronic hepatitis B in Chinese migrants: results of a project offering on-site testing in Rotterdam, The Netherlands. *Journal of Hepatology*, 57:1171-1176.  [Vu LH](http://www.ncbi.nlm.nih.gov/pubmed?term=Vu%20LH%5BAuthor%5D&cauthor=true&cauthor_uid=21159695), [Gu Z](http://www.ncbi.nlm.nih.gov/pubmed?term=Gu%20Z%5BAuthor%5D&cauthor=true&cauthor_uid=21159695), [Walton J](http://www.ncbi.nlm.nih.gov/pubmed?term=Walton%20J%5BAuthor%5D&cauthor=true&cauthor_uid=21159695), [Peet A](http://www.ncbi.nlm.nih.gov/pubmed?term=Peet%20A%5BAuthor%5D&cauthor=true&cauthor_uid=21159695), [Dean J](http://www.ncbi.nlm.nih.gov/pubmed?term=Dean%20J%5BAuthor%5D&cauthor=true&cauthor_uid=21159695), [Dunne MP](http://www.ncbi.nlm.nih.gov/pubmed?term=Dunne%20MP%5BAuthor%5D&cauthor=true&cauthor_uid=21159695), [Debattista J](http://www.ncbi.nlm.nih.gov/pubmed?term=Debattista%20J%5BAuthor%5D&cauthor=true&cauthor_uid=21159695). (2012). Hepatitis B knowledge, testing, and vaccination among Chinese and Vietnamese adults in Australia. *Asia-Pacific Journal of Public Health,* 24(2): 374-384.  Wiecha, J. M. (1999). Differences in knowledge of hepatitis B among Vietnamese, African-American, Hispanic, and white adolescents in Worcester, Massachusetts. *Pediatrics*, 104 (Supplement 6):1212-1216.  Wallace, J., McNally, S., Richmond, J., Hajarizadeh, B., & Pitts, M. (2011). Managing chronic hepatitis B: A qualitative study exploring the perspectives of people living with chronic hepatitis B in Australia. *BMC Research Notes*, *4*(1): 45.  Wu, H., Yim, C., Chan, A., Ho M, & Heathcote, J. (2009). Sociocultural factors that potentially affect the institution of prevention and treatment strategies for prevention of hepatitis B in Chinese Canadians. *Canadian Journal of Gastroenterology*, 23(1): 31-36.  Wu, C. A., Lin, S. Y., So, S. K., & Chang, E. T. (2007). Hepatitis B and liver cancer knowledge and preventive practices among Asian Americans in the San Francisco Bay Area, California. *Asian Pacific Journal of Cancer Prevention*, *8*(1):127-34.  Xiong, M., Nguyen, R.H.N., Staryer, L., Chathnaouvong, S., & Yuan, J-M. (2013). Knowledge and behaviours toward hepatitis B and the hepatitis B vaccine in the Laotian Community in Minnesota. *Journal of Immigrant and Minority Health*, 15 (4): 771-778. |
| --- |

*Supplementary Table 2: A Summary of hepatitis B knowledge*

| Author | Bastani *et al.* (2007) | Butler *et al.* (2005) | | Carabez, *et al.* 2014 | Cheung *et al.* (2005) | Coronado *et al.* (2007) | Cotler *et al.* (2012) | Hislop e*t al*. (2007) | Hwang *et al.* (2008) |
| --- | --- | --- | --- | --- | --- | --- | --- | --- | --- |
| Study participants / sample (female) | 141 (71) | 20 (12)  15-18yrs | 45 (30)  19-25yrs | 154(39) | 1008 (554) | 430 (224) | 201 (majority) | 504 (287) | 251 (138)  >18yrs |
| Ethnicity | Korean | Hmong | Hmong | Mixed Asians | Mixed Asian | Chinese | Chinese | Chinese | Vietnamese |
| Study population / recruitment | Korean Christian Churches & Korean serving primary care clinic | Lao Family Organization’s Family Supportive Service for young adults programme or roster of Hmong taken form Fresno City phone book | | Convenience sample of Asian Americans who self-reported HBV infection | A random selection of shoppers of two large Asian commercial centres in Richmond, BC. | Population-based sample through households | Adults presenting for routine care to an internal medicine practice | Random section among Chinese residents in Vancouver | A random sample of Vietnamese American students |
| Study location | Loss Angeles, USA | California, USA | | San Francisco, USA | Vancouver, Canada | Seattle, USA | Chicago, USA | Vancouver, Canada | Houston, USA |
| Aim | To understand hep B serologic testing and vaccination rates and associated knowledge. | Hepatitis B knowledge among the Hmong Youth and implications for liver cancer prevention strategies | | Explore knowledge, fears, and follow-up care among this with chronic HBV | Evaluate degree of concern for and knowledge of HBV in this high-risk community. | Examine factors associated with HBV testing | Develop and validate a HBV stigma scale and evaluate HBV stigma | Determine HBV testing, vaccination, and knowledge | Collect information on knowledge and behaviours related to HBV |
| Awareness of HBV | 85 | - | - | - | 68 | - | - | - | - |
| HBV can be spread by someone who looks healthy | 81 | - | - | - | - | 80 | 50 | 80 | - |
| ***Mode of transmission***  During sexual intercourse (YES) | 64 | 50 | 49 | 79 | - | 56 | 60 | 65 | 66 |
| Shaking hands (No) | - | - | - | - | - | - | - | - | - |
| Holding hands (NO) | - | - |  | - |  | - | - |  | - |
| During child birth (YES) | 79 | 55 | 56 | 77 | - | 76 | 91 | - | 59 |
| Sharing toothbrush (YES) | 71 | 45 | 33 | 70* | - | - | - | - | 45* |
| Sharing use of needles (YES) | - | - | - | - | - | - | - | - | 73 |
| When intravenous drug users share a needle (YES) | - | - | - | 69 |  |  |  |  | 59 |
| Sharing razor blades (YES) | - | - | - | - | - | - | - | 68 | - |
| Through blood / blood products (YES) | - | - | - | 75 | - | - | 90 | - | 65 |
| Sharing eating utensils (NO) | 62*** | - | - | - | - | - | 57** | 89** | - |
| Eating food prepared by an infected person (NO) | - | - | - | - | - | - | - | 76** | - |
| Sharing food and drinks with an infected person (NO) | - |  | - | - | - | - | - | - | - |
| Eating food that has been pre-chewed by an infected person (YES) | - | -- | - | - | - | - | - | - | - |
| Being coughed / sneezed on by an infected person (NO) | - | - | - | - | - | - | - | - | - |
| ***Prevention of transmission***  HBV screening and / or testing (YES) | - | - | - | - | - | - | 94 | - | - |
| Vaccination (YES) | 62 | 70 | 67 | 69 | 65 | 65 | 95 | - | 81 |
| ***Sequelae of infection***  HBV is a lifelong infection (/ chronic) (YES) | 36 | - | - | - | - | 38 | 75 | 45 | - |
| HBV can cause liver cirrhosis (YES) | - | - | - | 83 | 40 | - | 98 | 83 | 81 |
| HBV can cause liver cancer (YES) | 75 | 65 | 60 | 83 | 39 | 71 | 92 | 81 | 70 |
| People can die from HBV (YES) | 79 | - | - | 55 | - | - | - | - | - |
| Those who are infected are sometimes avoided by others | 38 | - | - | - | - | 54 | - | - | - |
| ***Treatments for HBV*** |  |  |  |  |  |  |  |  |  |
| HBV can be treated / cured (NO) | - | 50 | 44 | 40 | - | - | - | - | 66** |
| There are treatments / medicines for HBV | - | 86 | - | - | - | - | 84 | - |  |

*Combined sharing toothbrush and razor. **Original answer by authors was “no”. Figures were rounded to the whole number. Data were presented as % of correct answer for each item. ***Includes sharing food.

*Supplementary Table 2: A Summary of hepatitis B knowledge*

| Author | Kue and Thorbun (2013) | Lee *et al.* (2007) | | | Li *et al*. (2012) | Ma *et al.* (2007) | Ma *et al.* (2008) | Maxwell *et al.* (2012) | | | | | | |
| --- | --- | --- | --- | --- | --- | --- | --- | --- | --- | --- | --- | --- | --- | --- |
| Study participants / sample (female) | 39(44) | 109 (81)  city A | 171 (93)  city B | | 103 (238) | 256 (168) | 429 (264) | 653 (366) | | 260 (156) | | 493 (320) | 329 (171) | |
| Ethnicity | Hmong | Korean | Korean | | Chinese | Vietnamese | Chinese | Vietnamese | | Hmong | | Korean | Cambodian | |
| Study population / recruitment | A convenience sampling at community locations, community meetings, gatherings and activities. | A convenience sampling of Korean American households through five Korean American churches. | | | General physician office & ESL class | Cluster sampling from Vietnamese voluntary organisations | Cluster sampling from community organisations | Cluster sample from eligible households | | Cluster sample from eligible households | | Cluster sampling of Korean Churches | Cluster sampling from households | |
| Study location | Oregon, USA | Rocky Mountain, USA | | | Toronto, Canada | Philadelphia & New Jersey, USA | New York City, USA | Greater Washington, USA | | Greater Sacramento, USA | | Los Angeles, USA | Greater Seattle, USA | |
| Aims | knowledge of hepatitis B and screening and vaccination behaviour | Assess knowledge and attitude towards HBV , and determine socio-demographic correlates of HBV knowledge | | | Understand factors that influence hep B screening | Measure knowledge related to HBV screening and vaccination | Measure the knowledge, and barriers of HBV screening and vaccination | Examine the correlates of HBV knowledge and the similarities and differences in this across four Asian American groups. | | | | | | |
| Awareness of HBV | 90 | - | | - | - | 46 | - | 72 | 45 | | 79 | | | 66 |
| HBV can be spread by someone who looks healthy |  | - | | - | - | - | - | - | - | | - | | | - |
| ***Mode of transmission***  During sexual intercourse (YES) | 60 | 19 | | 24 | 66 | 23 | 40 | - | - | | - | | | - |
| Shaking hands (No) | - | - | | - | - | 9 | - | - |  | |  | | |  |
| Holding hands (NO) | 89 | - | | - | - | - | - | - |  | |  | | |  |
| During child birth (YES) | 81 | - | | 34 | 76 | 62 | - | 62 | - | | - | | | - |
| Heredity (NO) |  | 44 | | 44 |  |  |  |  |  | |  | | |  |
| Sharing toothbrush (YES) | 59 | - | | - | - | 42 | 52 | - | - | | - | | | - |
| Sharing use of needles (YES) | - | - | | - | - | 50 | 52 | - | - | | - | | | - |
| When intravenous drug users share a needle (YES) | 85 | - | | - | - | - | - | - | - | | - | | | - |
| Sharing razors with an infected person (YES) | 55 | - | | - | - | - | - | - | - | | - | | | - |
| Through blood or blood products (YES) | - | - | | - | - | - | - | - | - | | - | | | - |
| Sharing eating utensils (NO) | 52 | 18 | | 14 | - | 69 | - | - | - | | - | | | - |
| Eating food prepared by an infected person (NO) | 55 | - | | - | - | 29 | 67 | - | - | | - | | | - |
| Sharing food and drinks with an infected person (NO) | - | - | | - | 29 | - | - | - | - | | - | | | - |
| Eating food that has been pre-chewed by an infected person (YES) | 63 | - | | - | - | - | - | - | - | | - | | | - |
| Being coughed / sneezed on by an infected person (NO) | 46 | 48 | | 62 | - | - | - | - | - | | - | | | - |
| ***Prevention of transmission***  Hepatitis B screening and / or testing (YES) | - | - | | - | - | 32 | 62 | - | 68 | | 94 | | | - |
| Vaccination (YES) | - | - | | - | 96 | 71 | - | - | - | | - | | | - |
| ***Sequelae of infection***  HBV is a lifelong infection (/ chronic) (YES) | 68 | - | | - | - | - | - | 48 | - | | 51 | | | 57 |
| HBV can cause liver cirrhosis (YES) | - |  | |  | 85 | - | - | - |  | | - | | |  |
| HBV can cause liver cancer (YES) | 68 | - | | 55 | 85 | 65 | 72 | 75 | 59 | | 82 | | | 69 |
| People can die from HBV (YES) | 83 | - | | - | - | - | - |  |  | |  | | |  |
| Those who are infected are sometimes avoided by others | - | - | | - | - | - | - | 38 | 55 | | 47 | | | 70 |
| Treatments for HBV |  |  | |  |  |  |  |  |  | |  | | |  |
| HBV can be treated / cured (NO) | 46 | - | | - | 15 | - | - | - | - | | - | | | - |
| There are treatments / medicines for HBV | - | - | | - | 93 | - | - | 75 | - | | 83 | | | 62 |

*Combined sharing toothbrush and razor. **Original answer by authors was “no”. Figures were rounded to the whole number. Data were presented as % of response for each item. ***Includes sharing food.

*Supplementary Table 2: A Summary of hepatitis B knowledge*

| Author | Nguyen *et al.* (2010) | Takahashi *et al.* (2011) | Taylor *et al.* (2000) | Taylor *et al.* (2002) | Taylor *et al.* (2004) | Taylor, Choe, *et al.* (2005) | Taylor, Yasui, *et al.* 2005 | Taylor *et al.* (2006) | Taylor *et al.* (2009) |
| --- | --- | --- | --- | --- | --- | --- | --- | --- | --- |
| Study participants / sample (female) | 1704 (954) | 297 (163) | 75 (32) | (403) | 345 | 715 (370) | (370) | 395 (211) | 111 |
| Ethnicity | Vietnamese | Samoa (n=151) & Chamorro (n= 146) | Vietnamese | Cambodian | Vietnamese | Vietnamese | Vietnamese | Chinese | Cambodian |
| Study population / recruitment | Representative sample from eligible households | Convenience sample from community / church / cultural meetings | Randomly selected households | Representative sample from households | Random sample from households. | Random sample from households | Random sample from households | Random sample from households, | A sample identified from households. |
| Study location | California and Washington, DC, USA | California, USA | Seattle, USA | Seattle, USA | Seattle, USA | Seattle, USA | Seattle, USA | Seattle, USA | Seattle, USA |
| Aims | Determine factors associated with HBV testing | Measure HBV knowledge, screening and vaccination. | Examine HBV knowledge | Hepatitis B knowledge and practices | Examine factors associated with HBV testing | Describe awareness & knowledge of hepatitis, and levels of testing. | Examine factors associated with previous HBV testing | Describe HBV knowledge, testing, and vaccination levels. | Describe hepatitis B knowledge, testing and vaccination levels. |
| Awareness of HBV | - | 63 | 67 | 56 | 76 | 85 | - | - | 64 |
| HBV can be spread by someone who looks healthy | 69 | - | 58 | 23 | 81 | 78 | 75 | 79 | 43 |
| ***Mode of transmission***  During sexual intercourse (YES) | 54 | 63 | 55 | 48 | 71 | 69 | 68 | 54 | 46 |
| Shaking hands (No) | - | - | - | - | - | - | - | - | - |
| Holding hands (NO) | - | - | 70 | 69 | - | 75 | - | - | - |
| During child birth (YES) | 77 | 38 | - | - | 81 | 83 | 85 | 70 | 59 |
| Heredity (NO) |  | 30 | - | - | - | - | - | - | - |
| Sharing toothbrush with an infected person (YES) | 68 | - | 67 | 69 | - | 72 | 77 | - | 53 |
| Sharing use of needles (YES) | 85 | 63 | - | - | - | - | - | - | - |
| When intravenous drug users share a needle (YES) | - | - | 75 | - | - | - | - |  | - |
| Sharing razors with an infected person (YES) | - | - | 55 | - | - | 63 | - | 55 | - |
| Through blood or blood products (YES) | - | 61 | - | - | - | - | - | - | - |
| Sharing eating utensils (NO) | 33*** | 21 | - |  | - | - | - | 16 | - |
| Eating food prepared by an infected person (NO) | - | - | 21 | 24 | - | 36 | - | 23 | 28** |
| Sharing food and drinks with an infected person (NO) | - | 26 | - | - | - | - | - | - | - |
| Eating food that has been pre-chewed by an infected person (YES) | - | - | 71 | 67 | - | 71 | - | - | - |
| Being coughing and sneezing (NO) | 48 | - | 25 | 11 | - | 31 | - | 32 | 32** |
| ***Prevention of transmission***  Hepatitis B screening and / testing (YES) | - | - | - | - | - | - |  | - | - |
| Vaccination | - | - | - | - | - | - | - | - | - |
| ***Sequelae of infection***  HBV is a lifelong infection (/chronic) (YES) | 53 |  | 38 | 24 | 43 | - | 45 | 37 | 52 |
| HBV can cause liver cirrhosis (YES) | - | - | - |  | - | - |  | 75 | - |
| HBV can cause liver cancer (YES) | 81 | - | 63 | 54 | 83 | - | 83 | 73 | 67 |
| People can die from HBV (YES) | 92 | - | 80 | 72 | 93 | - | 91 | - | - |
| Those who are infected are sometimes avoided by others | 39 | - | - | - | 39 | - | - | - | - |
| ***Treatment for HBV*** |  |  |  |  |  |  |  |  |  |
| HBV can be treated / cured (NO) | 85** | - | 88 | 15 | - | - | - | - | - |
| There are treatments / medicines for HBV (YES) | - | - | - | - | 96 | - | - | - | 52 |

*Combined sharing toothbrush and razor. **Original answer by authors was “no”. Figures were rounded to the whole number. Data were presented as % of correct answer for each item.

*Supplementary Table 2: A Summary of hepatitis B knowledge*

| Author | Taylor *et al.* (2011) | Thompson *et al.* (2002) | Thompson *et al*. (2003) | van der Veen *et al.* (2010) | Veldhuijzen *et al*. (2012) | Vu *et al.* (2012) | | | | Wu *et al.* (2007) | Wu *et al.* (2009) | Xiong *et al.* 2013 |
| --- | --- | --- | --- | --- | --- | --- | --- | --- | --- | --- | --- | --- |
| Study participants / sample (female) | 667 (367) | (124) | (147) | 355 (192) | 285 (185) | 442 (261) | 433 (207) | | | 199 (105) | 204 (76) | 167 (91) |
| Ethnicity | Cambodian | Chinese | Chinese | Turkish-Dutch | Chinese | Chinese | Vietnamese | | | Chinese | Chinese | Laotians |
| Study population / recruitment | Random sample from households. | A random sample of households | A random sample of households | A sample was drawn from municipal administration. | A convenience sample of targeted population at outreach locations | Convenience sampling at 6 Chinese communities and 1 Vietnamese community settings | | | | Recruitment from community cultural events & meetings, Chinese language schools. | Patients CHB recruited at physician offices and Hospital liver clinic. | Convenience sample of Laotians who had utilised Lao Assistance Center |
| Study location | Seattle, USA | Seattle, USA | Vancouver, Canada | Rotterdam, Netherlands | Rotterdam, Netherlands | Brisbane, Australia | | | | San Francisco, USA | Toronto, Canada | Minneapolis / St. Paul, USA |
| Aims | Examine HBV awareness, knowledge & testing, and vaccination levels. | Determine HBV knowledge and practices | HBV knowledge and practices. | Determine levels of knowledge and awareness of HBV. | Assessment of knowledge and awareness before and after awareness campaign. | Assess the current state in knowledge and preventive vaccination and testing | | | | Determine HBV and liver cancer knowledge and preventive practices | Identify barriers to care for people with chronic HBV | Assess knowledge and behaviours related to HBV and vaccine |
| Awareness of HBV | 78 | - | 85 | - | - | - | | | - | 90 | - | 42 |
| HBV can be spread by someone who looks healthy | - | 48 | 68 | 54 | 60 | 45 | | | 59 | - | 83 | 54 |
| ***Mode of transmission***  During sexual intercourse (YES) | 72 | 48 | 56 | 53 | 43 | 50 | | | 54 | 51 | 80 | 20 |
| Shaking hands (NO) | - | - | - | - | - | 73 | | | 75 | - | - | - |
| Holding hands (NO) | 76 | 58 | 71 | - | - | - | | | - | - | - | - |
| During child birth (YES) | 69 | - | - | 54 | 58 | 76 | | | 67 | 59 | 85 | - |
| Sharing toothbrush (YES) | - | 68 | 86 | - | - | 72* | | | 66* | 56* |  | - |
| Sharing use of needles (YES) | 83 | - | - | - | - | - | | | - | 85 | - | 18 |
| When intravenous drug users share a needle (YES) | 92 | - | - | - | - | - | | | - | 59 | - |  |
| Sharing razor blades (YES) |  | - | - | - | - | - | | | - | 56 | - | - |
| Through blood / blood products (YES) | 80 | - | - | - | - | - | | | - | 63 | - | - |
| Sharing eating utensils (NO) | - | - | - | - | - | - | | | - | - | - | - |
| Eating food prepared by an infected person (NO) | 21 | 21 | 41 | - | - | - | | | - | - | - | - |
| Sharing food and drinks with an infected person (NO) | 33 | - | - | - | - | - | | | - | - | 48 | - |
| Eating food that has been pre-chewed by an infected person (YES) | - | 69 | 82 | - | - | - | | | - | - | - | - |
| Being coughed / sneezed on by an infected person (NO) | - | 15 | 25 | - | - | - | | | - | - | - | 7 |
| ***Prevention of transmission***  Hepatitis B screening and or / testing (YES) | 29 | - | - | - | - | - | | | - | - | - | - |
| Vaccination (YES) | - | - | - | 54 | - | - | | | - | 74 | 86 | 89 |
| ***Sequelae of infection***  HBV is a lifelong infection (/ chronic) (YES) | - | 27 | 39 | 44 | 53 | 51 | | | 41 | - | - | 73 |
| HBV can cause liver cirrhosis (YES) | - | - | - | - | - | - | | | - | 83 | 83 | - |
| HBV can cause liver cancer (YES) | - | 46 | 61 | 25 | 50 | 60 | | | 68 | 78 | 88 | 81 |
| People can die from HBV (YES) | - | 57 | 77 | 36 | 47 | - | | | - | 64 | - | 78 |
| Those who are infected are sometimes avoided by others | - | - | - | - | - | - | | | - | - | - | - |
| Treatment | - |  |  |  |  |  | | |  |  |  |  |
| HBV can be treated / cured (NO) |  | 18 | 25 | - | - | - | | - | | - | 57 | 28 |
| There are treatments / medicines for HBV (YES) | - | - | - | - | - | 69 | | 60 | | - | 63 | 44 |
|  | - |  |  |  |  |  | |  | |  |  |  |

*Combined sharing toothbrush and razor. **Original answer by authors was “no”. Figures were rounded to the whole number. Data were presented as % of correct answer for each item.

*Supplementary Table 3: Other quantitative / survey studies: A summary of hepatitis B and /or C knowledge*

| **Study** | **Location** | **Ethnicity** | **Study participants / recruitment** | **Study participants / sample *(female)*** | **Study focus & aims** | **Summary of outcomes** |
| --- | --- | --- | --- | --- | --- | --- |
| Caruana *et al.* (2005) | Melbourne, Australia | Laotians & Cambodians | Convenience sample of participants recruited through general practitioner (GP) practices | Laotians (n=95 (51))  Cambodians (n=234 (126)) | Focus: HBV and HCV  Aim: To assess the knowledge about HBV and HCV and estimate the seroprevalence markers. | *Laotians:* 74% had heard of ‘hepatitis’, a result that was not influenced by gender, age group or year of arrival in Australia (*p*=0.88, 0.13 and 0.75, respectively). 61% did not know of any symptoms or complications (66%) associated with hepatitis. 50% of those who had heard of hepatitis knew specifically of HBV and 47% knew of HCV. 44% and 27% of those who had heard of HBV and HCV respectively could identify possible modes of transmission.  *Cambodians*: 94% had heard of ‘hepatitis’, and this was not dependent on age group, gender, or year of arrival in Australia (*p=*0.62, 0.30 and 0.90, respectively). There was a significant association between knowing about hepatitis and previous exposure to HBV (*p=*0.01), which was not seen among those exposed to HCV (*p=*0.76). 53% had heard specifically of hepatitis A, B and C but 67% were unsure of the possible transmission pathways for these viruses. |
| Dev *et al.* (2004) | Melbourne, Australia | Southeast Asians (SEA) | Convenience sample of HCV patients attending a liver clinic | Caucasians (n=90)    SEA (72, (34)) = Vietnamese (n=45), Cambodian (n=26), & Laotian (n=1) | Focus: HCV  Aim: To assess the risk factor profile, perceived risk factors, knowledge of risk factors and methods to minimize risk. | South East Asian (SEA) Patients: *Routes / methods of HCV transmission* were blood (89%); food (20%); water, alcohol, mosquitoes, and poor hygiene (11%). *Mode of acquisition:* Only 33% of the patients were able to identify the most probable modes of their acquisition of HCV (*P* < 0.0001). *Methods used to minimize transmission:* All SEA patients practiced universal precautions in dealing with blood spills and avoided sharing toothbrushes and razors. 36% of SEA patients separated cooking utensils, crockery and cutlery, soap and food for fear of transmitting HCV. All SEA who were current IDUs did not share needles and syringes, but did share other injecting paraphernalia. 72 % of SEA patients believed traditional medical practices such as coin rubbing, cupping, moxibustion and acupuncture did not carry an increased risk of HCV transmission. 15% thought that direct cuts to the skin with scalpel blades or glass carried no risk. 43% believed cosmetic tattooing of eyebrows and eyelids was not associated with transmission of HCV. Younger age was associated with a correct understanding of their own risk factor, whereas level of education and duration of liver clinic attendance were not. |
| Flores *et al.* (2012) | Loss Angeles, USA & Cuernavaca, Mexico | Latinos and Mexicans | Convenience sample from waiting room areas of clinics and community events or sites. | USA:(Mexican Americans (Latinos) (n=101(44))  Mexico: (n=125) | Focus: HBV & HCV  Aim: To contrast the liver disease risk factors, knowledge, and prevention practices. | Nearly 70% and 57% of respondents in Mexican Americans knew that infection with HBV and HCV is a risk factor for getting liver disease respectively. While 34% and 7% of the participants reported that a HBV and HCV vaccine exists respectively, more than three-fourths of the participants incorrectly reported that HBV and HCV can always be cured. Participants indicated that HBV or HCV could be transmitted through: blood transfusion (82%) and sexual intercourse (50%). 40% of participants indicated that HBV and HCV infection could last a lifetime. |
| Horwitz *et al.* (2010) | Sydney, Australia | Egyptians | Convenience sample from Coptic Churches, community centres & gatherings, social networks & multicultural health service. | 121 (57) | Focus: HCV  Aim: To explore the knowledge and awareness of hepatitis C, and attitudes towards people living with hepatitis C. | 80% of the sample had heard about HCV. The most common source of information on HCV was radio. HCV can be transmitted via: unsterile tattooing and body piercing (three-quarters of participants); through sharing needles and syringes (78%); unsterile unsterile vaccinations or medical procedures overseas (78%); mosquito bites (55%); and eating and drinking with infected person (55%). There is a vaccine against HCV (83%); there is no effective treatment for HCV (66%); and knew that many people had been cured of HCV in Australia (46%). Greater HCV knowledge was correlated with younger age, higher levels of education, employment, and favourable attitudes towards people living with HCV. Regression analysis revealed that younger age (Beta = -.299, p<.05), having heard about HCV (Beta = .47, p<.001), having had a blood test for hep C (Beat =.231, p<.01) and a favourable attitude towards people living with HCV (Beta =.210, p<0.01) were independently related to better HCV knowledge in the sample. |
| Ma *et al.* (2006) | New York City, USA | Chinese | Multi-stage cluster sampling from ten randomly selected Chinese community-based organizations. | 429 (264) | Focus: HBV  Aim: To measure the risk perceptions, barriers, and self-efficacy. | The statistically significant barriers to obtaining screening were: feeling well (34.6%), did not know where to get screened (12.5%), their doctor did not suggest it (17.7%), and not knowing if insurance plan covered HBV screenings or vaccinations (24.8%). The statistically significant barriers to obtaining vaccination were: no time (10.8%), did not know where to get screened (12.5%), and having a primary care provider (85.6%). The perceived benefits of screening included getting reassurance that they did not have HBV (44.5%) and self-efficacy (51.6%). Participants reported that if they discussed HBV with family members (62.8%), friends (64.8%), and their physician advised (49.7%) they would be more likely to go for screening or vaccination. The variables which inversely correlated with vaccination behaviour were: not having time, did not know a screening location, and not speaking the physician’s language. The variables which positively correlated with vaccination behaviour were having a primary care physician. |
| O'Connor *et al.* (2008) | Sydney, Australia | Vietnamese | A list of 100 most common Vietnamese family names matched against telephone numbers in electronic telephone book. | 499 | Focus: HBV&HCV  Aim: To describe hepatitis B knowledge and self-reported infection and risk behaviour. | Univariate predictors of hepatitis B knowledge were: being highly acculturated (*P* < 0*.*001); perceiving yourself to be in good health (*P* < 0*.*01); ever having been tested for HIV (*P* < 0*.*05); and knowing someone with HIV (*P* < 0*.*01). On multiple regression analysis, being highly acculturated (*P* < 0*.*001), ever having been tested for HIV (*P* < 0*.*001) and knowing someone with HIV (*P* < 0*.*0001) remained predictive. There was no significant difference in hepatitis B knowledge in those who had ever been infected with hepatitis B and diagnosed in the last year compared with those diagnosed previously.  Univariate predictors of hepatitis C knowledge were: being highly acculturated (*P* < 0.01); being employed full time (*P* < 0.05); ever injecting drugs (*P* < 0.05); and being vaccinated for hepatitis B (*P* < 0.001). On multiple regression analysis being highly acculturated (*P* < 0.001), ever injecting drugs (*P* < 0.05) and being vaccinated for hepatitis B (*P* < 0.001) remained predictive. |
| Shiau *et al.* (2012) | San Francisco, USA | Chinese, Filipino, Japanese, Vietnamese, Taiwanese, Korean, & other | *Street intercept interviews*: A convenient sample recruited via street in purposively selected sites *Telephone survey:* A sample of telephone numbers associated with Asian surnames. | 306 (194)  Chinese (n=264), Filipino (n=14), Japanese (n=11), Vietnamese (n=10), Taiwanese (n=3), Korean (n=2), other (n=5) | Focus: HBV  Aim: To determine level of awareness about hepatitis B and evaluate the impact of the “Be a Hero” media campaign. | 67% had heard of HBV. Knowledge: asymptomatic individuals can transmit the disease to others (60%), HBV is more easily spread than HIV (27%), HBV can cause liver cancer (63%), HBV can cause death (83%), HBV infection can be lifelong (38%), HBV is incurable (22%). 41% did not think that there was anything they could to protect themselves, and their families against infection, and 28% had not heard of the HBV vaccine. |
| Wiecha (1999) | Massachusetts, USA | Vietnamese | A convenience sample of all students at 2 public high schools and 2 public middle schools. | 2816 Vietnamese (n=226, no gender given) | Focus: HBV  Aims: To assess the level of knowledge HBV, and compare it to the knowledge of other adolescents of other races and ethnicities. | Adolescent knowledge of HBV and about risk of infection was low. Only 35.6% of Vietnamese respondents were more likely than were other students to know that HBV affects the liver. Only 13.7% were likely to correctly identify sex with an infected person as a risk factor for infection. Independent predictors of this knowledge were: white race; older age; attending high school versus middle school; having been taught about HBV in school; knowing the definition of HBV; reporting better grades; having a family member with HBV; and being more highly acculturated. |

*Supplementary Table 4: Qualitative studies: A summary of hepatitis B and /or C knowledge*

| **Study** | **Location** | **Ethnicity** | **Study participants / recruitment** | **Study method** | | **Participants / sample *(female)*** | **Aims** | **Summary of outcomes** |
| --- | --- | --- | --- | --- | --- | --- | --- | --- |
| Burke *et al.* (2004) | Seattle, USA | Vietnamese | A convenient sample recruited by community members, research assistants, and community advisors. | Qualitative - 25 open-ended in-depth interviews and 6 focus groups (8 of participants of equal gender in each). | | 47 (23) | Focus: HBV and liver cancer.  Aims: To identify cultural factors influencing HBV knowledge. | Hepatitis B is caused by ‘damaged liver’ as result of harmful foods, environmental pollutants and toxins, physical deprivation, incarceration and poverty; and hormones. Beliefs about HBV cause and transmission are influenced by traditional Vietnamese and traditional Chinese medicine theory, indigenous values placed on health and positive attitude, and personal experiences and socio‐historical circumstances. Transmitted by mother to child through birth, sharing earrings; sharing foods and drinks; sharing eating utensils, |
| Burke *et al.* (2011) | Seattle, USA | Cambodian | A convenient sample recruited through community coalition members and research assistants’ social networks. | Qualitative – 8 Focus group discussions segregated by age (20-39 & 40-64) and gender | | 97 (49) | Focus: Liver cancer and HBV  Aim: To generate peoples’ understanding of health and illness, HBV testing, HBV treatment, and vaccination. | HBV is understood as a liver disease. Symptoms associated with HBV are tough liver, swollen belly, and dysfunctional liver. Understanding of HBV disease course, transmission, prevention, and treatment are influenced by socio-cultural factors, historical circumstances and experiences in countries of origin, and resettlement and migration experience, humoral theories on Khmer medicine, and biomedicine. There is linear and progressive understanding of types of viral hepatitis infections where A progresses to B, then to C. And A being deadly, B not very good, and C okay. HBV is related to HIV/AIDS. Cause is associated with new foods, imbalance and disharmony in the body, migration and lifestyle changes, environment (remote conditions, poor sanitation and farming of livestock and chicken in Cambodia). There is lack of understanding of the around vaccinations. HBV causes liver cancer. Cold food could cure HBV, and Chinese medicine eases discomfort. |
| Chang *et al.* (2008) | San Francisco, USA | Chinese | A convenient sample from local community-based organisations, health centres, libraries, schools, and supermarkets. | Qualitative – six focus groups of 4-12 participants of mixed gender, stratified by language (Cantonese, Mandarin, and English). | | 47 (22) | Focus: HBV and liver cancer  Aim: To inform the development of community-based programmes to increase hepatitis B and liver cancer awareness and prevention. | Factors that motivated people to get tested included peace of mind, prevention of transmission to others, informed decision-making ability, convenience, and pre-vaccination screening. Primary motivations for hepatitis B vaccination were protection of future health and avoidance of hepatitis B. People were discouraged from testing or vaccination by: costs; lack of health insurance; fear of side effects; worries about reliability and efficacy; poor patient-doctor communication; reliance of professional opinion; apparent good health; inconvenience; and personal preference. |
| Chen *et al.* (2006) | Seattle, USA & Vancouver, Canada | Chinese | Purposive recruitment by staff of local community health clinics and service organisations through social networks | Qualitative - Semi-structured and in-depth interviews | | 40 (22)  Seattle – n=20  Vancouver – n=20 | Focus: HBV  Aim: To learn about the hepatitis prevention behaviour, along with their knowledge, beliefs, and perceptions with regard to hepatitis, screening, and vaccination. | General lack of accurate knowledge of viral hepatitis and confusing of various types. Cause of HBV associated with ‘damaged liver’, emotional imbalance, harmful food, and contact with infected persons. HBV is contagious and causes liver cancer. Liver can be protected through use of Chinese health beliefs (herbal medicine, stress-free mind, strengthening body’s defences, and getting adequate sleep). HBV can be prevented through good hygiene and avoiding harmful food. HBV can be treated with vaccination. |
| Choe *et al.* (2005) | Washington, USA | Korean | Sample recruited from churches, and community-based organizations | Qualitative – 30 semi-structured interviews and two focus groups of 18, mixed gender | | Interviews - 30 (15),  Focus group - 18 (13) | Focus: HBV and liver cancer  Aim: To investigate hepatitis and liver cancer prevention, behaviour, and beliefs | HBV is caused by contamination of food sources. HBV transmitted by sharing of food utensils, exposure to blood, and sexual contact. Alcohol can cause long-term liver sequelae, and combined with HBV increases the risk of liver cancer. Prevention of HBV is altering eating habits, preparing meals carefully, reducing alcohol consumption, regular walking, and reducing stress. |
| Coupland & Maher (2010) | Southwest Sidney, Australia | Cambodia, Lao, and Vietnamese | Theoretical and snowball sampling of IDUs through street and peer networks. | | Ethnography | 72 (13)  Cambodians -n=14; Laos - n=17; and Vietnamese -n=41. | Focus: HCV  Aim: To explore factors influencing treatment uptake by three marginalised ethnic minority IDUs and their influence on prevention and treatment of hepatitis C. | HCV is transmitted via sharing injecting equipment, tattoos, direct contact with open wounds, or ‘cuts’ of people with HCV, sharing razors, sharing drug injecting paraphernalia (like spoons, swabs, water, tourniquets, filters), and ‘blood-to-blood’ transmission’. Shame is associated with HCV especially because of its perceived link with “junkie” behaviour, “carelessness” and lack of hygiene. HCV prevention and treatment-seeking were predominantly influenced by a cultural context heavily shaped by the stigma associated with injecting drug use and the perceived marginalisation, which led to them positioning themselves outside of the public health systems thus viewing advice on HCV prevention and treatment as irrelevant or out of reach. HCV infection was experienced as a marker of "spoiled identity"**,** identifying an individual as an injecting drug user. Racism, stigma, discrimination, and practices and policies within health services were seen as barriers to candidacy for treatment. |
| Ho *et al.* (2008) | South West Sydney, Australia | Vietnamese | Recruitment through a mix of snowball, and theoretical sampling drawing on street and social networks. | Ethnographic methodologies: observational and in-depth interviews | | 58 (8) | Focus: blood-borne viral infections  Aim: To explore the influence of cultural beliefs and practices on vulnerability to blood-borne viral infections among those who have injected drugs | HBV and HCV is transmitted through mosquitoes, saliva, & sharing injecting needles and syringes with strangers, but not with friends, which is seen as ‘less risky’, ‘normal’, and ‘expected’. The contraction of HBV is also explained within the context of fate – some sort of inevitability – linked with the philosophy of Buddhism. Participants were not aware of efficacy of, and availability of antiviral treatment. Stigma and discrimination was associated with viral infections mainly due to its link with illicit drugs. This, and perceived lack of confidentiality and being judged by practitioners limited disclosure of IDU practice, and was a barrier to hospital access. |
| Hwang *et al.* (2012) | Houston, USA | Chinese, Korean, & Vietnamese | Recruitment by emails, direct invitations, through a list of business, organizations, and religious leaders and board members, word of mouth, bilingual fliers and face-to-face solicitations. | Qualitative - 12 focus groups, participants stratified by ethnicity | | Chinese – 39 (18)  Korean – 32 (15)  Vietnamese – 37 (19) | Focus: HBV  Aim: To explore in-depth HBV-related healthcare attitudes of Chinese, Korean and Vietnamese communities with different levels of acculturation. | Eating certain food, poor hygiene, fatigue / stress & drinking alcohol associated with HBV aetiology. Prevention is perceived to be through improving on personal hygiene, proper nutrition, exercise, and holistic methods (resting, using alternative medicine & living healthily). Treatment with Western medicine (doctors) first and complementary / alternative medicine later, having vacation, eating vegetables, eating regularly and working out regularly. |
| Philbin *et al.* (2012) | Maryland, USA | Chinese, Korean & Vietnamese | Recruitment through many sources: flyers; community events; newspapers; e-mails; staff members’ networks. | Qualitative – 8 focus Group discussions stratified by age: 4 were of those <25 yrs.; 6 were of ≥25year. | | Mixed gender (58 (31)): Chinese – n=20, Korean – n= 19, and Vietnamese – n= 19 | Focus: HBV and liver cancer  Aim: To identify perceptions, understanding and barriers regarding hepatitis, screening and liver cancer prevention. | Awareness of HBV influenced by generation. The awareness of members of older and younger generations was framed in terms of technology and information access. Youth perceived as having increased access to medicine, information, and knowledge. Other themes are: perceived lack of susceptibility to HBV and liver cancer; the role of fate in developing HBV and liver cancer; stress as a risk factor for developing HBV and liver cancer. Barriers to prevention mentioned were culture, complexity of health care systems, and stigma. |
| van der Veen *et al.* (2009) | Rotterdam, Netherlands | Turkish | Recruitment through Islamic organization who contacted 2 different Turkish men and women associations. | Qualitative – 8 focus groups, stratified by generation and gender | | 54 (31) | Focus: HBV  AIM: Investigate the socio-cultural determinants associated with hepatitis B screening | HBV perceived as an STD, related to HIV/AIDS, acquired through extra- or pre-marital sexual contacts. Getting HBV is also due to not living according to the rules of Islamic religion under the concept of cleanliness – living in the halal (lawful) way. Girls would find it hard to speak about HBV with their parents because of the doctrine of honour related to sexual behaviour of women in the family. HBV could be prevented by following Quran (Muslims are obliged to care for their body, in order to be able to return it to Allah in unblemished state, keeping once body pure). HBV infection might be an impediment to getting married. |
| Wallace *et al.* (2011) | Victoria & South Australia, Australia | Mixed group of migrants:  Vietnam ,  China, Cambodia and Afghanistan | Purposive sampling of people with chronic HBV through public hospitals’ liver clinic, Hepatitis C and community NGOs and professional networks of investigators. | Qualitative - semi-structured interviews; four focus group discussions | | Interviews: 20 (7): Vietnam (n=6); China (n=5), Cambodia (n=3) & Afghanistan (n=2)  & 4 focus group with 40 staff and volunteers | Focus: chronic HBV  Aim: To record how people with chronic HBV respond to their infection. | People with chronic HBV had a poor understanding of their infection. HBV is associated with poor sanitation. People had a linear understanding of viral hepatitis as existing at three levels: hepatitis A, hepatitis B and hepatitis C with hepatitis C being the worse. Participants acknowledged being tested without consent, and received little or no information pre-and-post testing / diagnosis. Participants had reservations with the capacity of health professionals to respond their illness effectively. The study provides a more nuanced perspective on the confusion about relationships between hepatitis viruses and HIV, treatment, and transmission risks. |

*Supplementary Table 5: Mixed Methods studies: A summary of hepatitis B knowledge*

| **Study** | **Location** | **Ethnicity** | **Study participants / recruitment** | **Study method** | **Participants / sample *(female)*** | **Aims** | **Summary of outcomes** |
| --- | --- | --- | --- | --- | --- | --- | --- |
| Kue and Thorburn (2013) | Oregon, USA | Hmong | Recruited through written and oral communication at community locations, community meetings, gatherings and activities. | Semi-structured in-depth interviews and Survey measures | 39 (44)  Out of this 7 were hepatitis B positive | Aim: Examine knowledge of hepatitis B and screening and vaccination behaviour | Low of knowledge of hepatitis B transmission, especially through sharing tooth brush and razors. Transmission knowledge was higher among younger participants, those bot in the US, and those who reported seeking preventive care. Transmission and sequelae knowledge was not associated with screening and vaccination behaviours. There was confusion about different types of hepatitis (A / B). While some participants found it easy to get vaccinated if whole family is involved, some lacked information on the testing process. Lack of information on vaccination. Those infected with hepatitis B did not have adequate understanding of their illness. Survey results confirmed qualitative findings. |

*Supplementary Table 6: Translation and application of an adapted PEN-3 model of analyses*

PEN-3 model was originally developed to situate culture at the centre of health-seeking behaviour in health promotion and disease prevention [45-47], and emphasises the meeting and working with beliefs of participants rather than only aiming to change them. Using an adapted PEN-3 model, we have categorised themes (table below) as: *perceptions* (knowledge, attitudes, and beliefs that contribute or hinder health-seeking behaviour), *enablers* (community and structural factors), and *nurturers* (factors in one’s social network that reinforce health behaviours). Secondly, we have analysed them as either ‘positive’, negative or neutral’ depending on their potential impact on health-seeking (such as seeking screening and / or treatment) behaviours. The guide indicates specific areas of interventions (based on key findings) for addressing the negative factors (attitudes and beliefs), and reinforcing the positive ones, in a way that might influence health-seeking behaviour of immigrants thus improving on uptake of screening, vaccination, treatment, and follow-up care. For instance, one of the facets of intervention would be on culturally-influenced and targeted public health education and awareness-raising to address inadequate knowledge, but also reinforce the positive contexts of culture and behaviours.

| **Key findings** |  | **Strategies and approaches for intervention** | |  |
| --- | --- | --- | --- | --- |
| **Perceptions** |  | **Negative / positive / neutral** | **Reinforce (+)** | **Revise (-)** |
|  | Inadequate knowledge of HBV and HCV risk and transmission factors | Negative | - Reinforce knowledge on these transmission risk factors: blood, premasticated food, sex, child birth, intravenous needles for illicit drug use and therapeutic injections , sharing personal effects (toothbrush, razors), invasive traditional healing practices, tattoos and body piercing, | - Provide culturally-adapted and appropriately-targeted education through mass media that focuses on risk and routes of transmission. - Revise the perception that transmission is possible through dirty / uncooked food, cough and nasal discharge, breast milk, and sharing of food. - Provide links to resources on information about HBV and BCV. - Revise knowledge that HBV caused by damaged or weak liver, emphasising that HBV and HVC are viral infections, but emphasise the link between liver disease / cancer and HBV and HCV infections. - Clarify confusion on various types of viral hepatitis. - Provide knowledge that particular groups of immigrants are at a high risk of infections |
|  | Inadequate knowledge of prevention of HBV and HVC infections | Negative | - Reinforce knowledge of prevention through vaccinations and screening | - Provide information on effective preventive practices. Revise people’s perceptions of ineffective preventive practices like not sharing food and utensils, avoiding intake contact with people, traditional medicines, and adopting healthy lifestyles and practices. |
|  | HBV and HCV Infection brings shame and stigma, and a barrier to employment and / or marriage | Negative |  | - Provide educational materials that include risk factors, clarify sexual and injecting drug transmission risk factors to reduce shame - Explain that a test, and treatment for those infected with HBV or HCV will result in better outcomes - Demonstrate that confidentiality will be ensured. - Clarify that people can still get employment and / or marry even if they infected. |
|  | Stoic and fatalistic attitudes towards health and illness and the notion that suffering and perseverance are admirable traits | Negative |  | - Emphasise individual responsibility for health, and encourage help-seeking behaviour and use survivors narratives of experience infections and care, emphasise on curability. |
|  | Inadequate knowledge about the availability of treatment available for HCV and HBV infections. | Negative |  | - Provide information on treatments for HCV and HBV. Clarify that there is no cure for HBV, though there is effective long-term treatment with regular check-ups to reduce risk of developing liver cancer. |
|  | Vaccines not perceived as primary means of HBV prevention | Negative |  | - Provide education and information on the importance of HBV vaccine |
|  | Poor knowledge of screening | Negative |  | - Provide adequate education and information on screening for HBV and HCV, and its importance (such as prevention of liver disease and spread of infections). - Provide knowledge of screening process and procedures |
|  | Poor knowledge of treatments available |  |  | - Provide education that that there are treatments for HCV and HBV that prevents those who are infected from getting liver disease and premature death. |
|  | HBV and HCV are chronic infections and cause liver disease, liver cancer, and death | Positive | - Reinforce knowledge | - Provide education and information resources on health implications of untreated HBV and HCV infections, emphasising the adverse and fatal sequelae of untreated viral infections. |
|  | Family and children are a source of motivation to be screened | Positive | - Reinforce importance of engaging in preventive practices (screening) and motivation to be healthy for self, and family. |  |
|  | Receptive to information and health education | Positive | - Reinforce health literacy by providing knowledge about risk factors as well as about screening, vaccination, and treatment | - Provide culturally-adapted and appropriately-targeted public health education. |
| **Enablers** | Trust in doctors and other healthcare providers as a source of health information and recommendations (for screening and vaccination) | Positive | - Doctors, other healthcare professionals and providers to actively recommend screening and vaccination to at-risk immigrant groups | - Decrease structural barriers to accessing doctors, and other healthcare providers. - Encourage doctors and other healthcare professionals to provide accurate health information to immigrants. - Provide training to heath care professionals to improve on their cultural sensitivity and competency - Address barriers of communication between professionals, healthcare providers and immigrants |
|  | Use of traditional healers for treatment and source of health information | Positive |  | - Health information on HBV and HCV should be provided to traditional healers (e.g. acupuncturists) - Healthcare system to form an alliance with traditional healers and community leaders. |
|  | People experience problems with booking for appointments, convenient appointment times, and long waiting lists.  Lack of health insurance & cost of test / vaccination prevent access. | Negative |  | - Provide adequate knowledge on cost issues (as some countries like the UK currently offer free access) - Promote knowledge of where to obtain test / vaccination - Provide services at appropriate times (making access to services flexible) and ensure simplify appointment booking system - Education on cost implications for vaccines and medications. |
|  | Difficulty on obtaining referrals and suitable appointments | Negative |  | - Healthcare providers to simply referral system (preferably have community based care) and have flexible appointments with shorter waiting lists. |
|  | Healthcare provider’s (and professionals’) lack of adequate information on HBC and HCV | Negative |  | - Healthcare professionals should be well equipped with knowledge to be able to provide adequate information on HBV and HCV during pre- and post-testing consultations that might improve the effectiveness of communication about risk, prevention, and care management. - Provide translators where language and cultural barriers could contribute to lack of communication and inadequate information giving. |
|  | Differences in health system from home country | Negative |  | - Provide education on healthcare systems |
|  | Reluctance to visit a doctor just for a test | Negative |  | - Add tests to routine blood tests, and target the whole family |
|  | Lack of provision of pre-and post-testing information | Negative |  | - Increase knowledge on screening process and procedures - Provide adequate and comprehensible information on test results |
|  | Personal experiences with screening and vaccination | Negative / Positive | - Reinforce positive experiences such as the need to know status; need to protect one’s self; and the benefit for whole family. |  |
|  | Fear of screening and screening results | Negative |  | - Clarify that screening is done through blood, and indicate ways of drawing blood - Provide information on the process of screening, and offer counselling to address fears of testing results |
|  | Screening without consent | Negative |  | - People should be provided with adequate information, and informed consent should be obtained before screening. |
|  | Complex healthcare (e.g. unclear process of access for screening, vaccination) | Negative |  | - Provide information on access to healthcare resources - Simplify the process of accessing care for screening, vaccination and treatment |
| **Nurturers** | Confusion around the process of screening | Negative |  | - Provide information on testing process, its importance on prevention and early detection of infection, and what the results mean (especially terminologies such as ‘antigen, ‘antibody’). |
|  | Confusion and uncertainty about the purpose and process of vaccination | Negative |  | - Provide education and adequate information on vaccination, its importance, efficacy, side-effects, cost, the number of shots needed and their frequency, to clear uncertainties around vaccinations. |
|  | Cultural beliefs against seeing a doctor (i.e. for a test or vaccination) when one is not ill | Negative |  | - Provide education to revise beliefs and stress importance of preventive healthcare |
|  | Positive perception of compulsory (actively recommended) testing and screening | Positive | - Have active policies and practices that promote routine targeting of at-risk immigrants |  |
|  | Perceived discrimination against those who are infected with HBV or HCV | Negative |  | - Educate immigrants focussing on transmission modes, preventability and treatability. |
|  | Close communal links among immigrant communities | Positive | - Provide education in culturally acceptable community settings |  |
|  | Motivation to keep families healthy and concern for family’s future | Positive | - Interventions should address the whole family as key family and friends can encourage help-seeking |  |
|  | Trust in family and friends ‘ recommendations | Positive | - Families should be used as entry points for interventions, rather than focussing on individuals only. Community networks (and those of friends) are important as pathways for reaching individuals - Health educational information (e.g. need for screening) to come from individuals who are perceived to be from similar socio-cultural and ethnic backgrounds or groups. | - Intervention programmes that target immigrants should be community-based |
|  | Cultural and Religious beliefs such as being clean and taking responsibility for one’s health, and health of family and community | Positive | - Programmes to embrace positive values of cultural / religious beliefs (such as disapproval of premarital and extra-marital sexual relationships, and encouragement of people to keep their bodies clean, and protect others from infection) reinforcing the necessity for testing and treatment. |  |
|  | Targeting whole family for intervention is more acceptable | Positive | - In closely knit communities, messages and interventions should target whole communities and families. |  |
